# The Role of Chronic Inflammatory Bone and Joint Disorders in the Pathogenesis and Progression of Alzheimer's Disease

**DOI:** 10.3389/fnagi.2020.583884

**Published:** 2020-12-07

**Authors:** Robert A. Culibrk, Mariah S. Hahn

**Affiliations:** Department of Biomedical Engineering, Rensselaer Polytechnic Institute, Troy, NY, United States

**Keywords:** Alzheimer's disease, osteoarthritis, osteoporosis, rheumatoid arthritis, inflammation, mesenchymal stem cells

## Abstract

Late-onset Alzheimer's Disease (LOAD) is a devastating neurodegenerative disorder that causes significant cognitive debilitation in tens of millions of patients worldwide. Throughout disease progression, abnormal secretase activity results in the aberrant cleavage and subsequent aggregation of neurotoxic Aβ plaques in the cerebral extracellular space and hyperphosphorylation and destabilization of structural tau proteins surrounding neuronal microtubules. Both pathologies ultimately incite the propagation of a disease-associated subset of microglia—the principle immune cells of the brain—characterized by preferentially pro-inflammatory cytokine secretion and inhibited AD substrate uptake capacity, which further contribute to neuronal degeneration. For decades, chronic neuroinflammation has been identified as one of the cardinal pathophysiological driving features of AD; however, despite a number of works postulating the underlying mechanisms of inflammation-mediated neurodegeneration, its pathogenesis and relation to the inception of cognitive impairment remain obscure. Moreover, the limited clinical success of treatments targeting specific pathological features in the central nervous system (CNS) illustrates the need to investigate alternative, more holistic approaches for ameliorating AD outcomes. Accumulating evidence suggests significant interplay between peripheral immune activity and blood-brain barrier permeability, microglial activation and proliferation, and AD-related cognitive decline. In this work, we review a narrow but significant subset of chronic peripheral inflammatory conditions, describe how these pathologies are associated with the preponderance of neuroinflammation, and posit that we may exploit peripheral immune processes to design interventional, preventative therapies for LOAD. We then provide a comprehensive overview of notable treatment paradigms that have demonstrated considerable merit toward treating these disorders.

## Introduction

### Canonical CNS Targets for AD Therapy

Pathological β-amyloid accumulations were among the earliest recorded physiological manifestations of AD (Tomlinson et al., 1970) and, along with neurofibrillary tangles, are considered a hallmark of AD-related neurodegeneration. It is therefore understandable that herculean efforts have been made in past decades to uncover their etiological contributions and evaluate whether interventions targeting plaque accumulations ameliorate patient outcomes. While numerous animal studies have demonstrated significant dose-dependent attenuation of Aβ accumulation when such therapies were administered to a variety of transgenic disease models (Wiessner et al., 2011; Eketjäll et al., 2016), these results were rarely—if ever—recapitulated in clinical trials (Holmes et al., 2008; Salloway et al., 2011; Egan et al., 2018).

Interest in tau-targeting therapies has drastically increased in recent years, in part due to the overall failure of Aβ-directed treatment paradigms. Under neurotypical conditions, tau proteins surround neuronal microtubules in an organized lattice, affording structural integrity and facilitating inter-neuronal nutrient transport (Vershinin et al., 2007). In AD, however, through a series of complex immunological and neurophysiological events that transpire well before the onset of cognitive impairment (Braak et al., 2006), tau proteins undergo a series of post-translational modifications—including and chiefly hyperphosphorylation. These not only disrupt their standard microstructure but also promote aggregation into fragments which both directly and indirectly incite neuronal necrosis (Gong and Iqbal, 2008). While tauopathies present as a heterogenous mixture of paired helical filaments, straight filaments, twisted ribbons and oligomeric aggregates in the AD brain, oligomeric tau has recently emerged as the current research focus, owing to its strong cytotoxic effect in preclinical models and its prominence in early stages of AD and mild cognitive impairment (MCI) (Mufson et al., 2014; Guerrero-Muñoz et al., 2015). Unfortunately, as with Aβ-targeting therapeutics, a variety of promising drugs and immune therapies designed to target tau protein modification, prevent tau aggregation, or promote phagocytosis of cytosolic tau have either produced modest or negligible clinical benefits, resulted in adverse effects, or demonstrated suboptimal long-term pharmacokinetics. The results of these recent clinical studies, representing a broad gamut of tau-targeting therapies, have been comprehensively reviewed elsewhere (Congdon and Sigurdsson, 2018).

Of unclear significance to the neurodegenerative cascade in the AD brain is the generation, activation, and proliferation of disease-associated microglia (DAMs). Despite the intrinsic phenotypic heterogeneity of microglia, DAMs are functionally and pathologically distinct from their neurotypical counterparts: they express significantly lower levels of genes related to microglial homeostasis (including a host of purinergic receptors) and express far greater levels of genes associated with AD risk, including Apolipoprotein E *(APOE)*, Lipoprotein lipase *(LPL)*, and Triggering Receptor on Myeloid Cells *2 (TREM2)* (Keren-Shaul et al., 2017; Ofengeim et al., 2017). Likely due to sustained neuroinflammation, microglia proximal to sites of neuronal necrosis or pathological protein aggregation transition to a semi-activated state, demonstrating abrogated expression of homeostatic regulatory genes and robustly upregulated chemotactic cytokine activity. A TREM2-mediated secondary activation event then occurs, wherein microglia are rendered incapable of phagocytizing AD substrates, develop a “frustrated” phenotype, and subsequently contribute to the secretion of neuroinflammatory factors (Michaud and Rivest, 2015; Kabba et al., 2018). It is yet uncertain whether the net effects of DAMs in the early- and late-stage AD brain are beneficial yet insufficient, or altogether detrimental. While microglia-mediated neuroinflammation has garnered tremendous interest in recent years, no specific microglia-targeting therapy has reached clinical trials at the time of this review.

The dysregulation of microglial behavior in late stage AD has recently been credited to a series of missense mutations in TREM2-encoding genes at various loci. TREM2 is a transmembrane glycoprotein commonly expressed on granulocytes and monocytes. Its primary function is the modulation of leukocytic function; specifically, immunocyte activation following antigen recognition (Martin and Delarasse, 2018). A mutation at exon 2 of *TREM2*—which encodes a substitution of histidine for arginine at index 47 (R47H)—has been shown to result in abrogated TREM2 signaling potential. This loss of function prevents effective microglial phagocytosis of AD substrates and is believed to be one of the main sources of pathogenic effects in the AD brain (Doens and Fernández, 2014). Microglia-mediated neuronal degradation could be attributed to the aggressive encircling of synaptic clefts when microglia carrying the mutated variant interact with AD substrates—an effect further intensified by the microglial spread of insoluble tau. Indeed, TREM2 missense has been implicated in the spread of tau aggregates via cyclical failed phagocytosis and subsequent exocytosis, irrespective of synaptic transmission (Colonna and Holtzmann, 2017). Clinical deficits associated with the R47H mutation are apparent: patients carrying the missense variant exhibited lower-than-average performance on a series of cognitive assessments, particularly those involving a series of temporal memory tasks (Jonsson et al., 2013). The observed decline implicates TREM2 missense in the early cognitive deterioration that results in AD and is in line with the prevailing hypothesis that the disease manifests in accelerated mental aging. Nonetheless, despite the abundance of correlative genome-wide and preclinical studies involving this key microglial receptor, the specific mechanisms by which TREM2 missense propagates in a neuro-degenerative pathology are poorly understood and methodologies for targeted therapeutic intervention have yet to be realized. Moreover, while *TREM2* research has undoubtedly offered new insight into the underlying pathological processes of AD, its mutation, along with mutations in Presenilin 1 (*PS1*), Presenilin 2 (*PS2*), and Amyloid precursor protein (*APP*) account for <5% of all AD cases (National Institute of Aging, 2011).

### Chronic Peripheral Inflammation—A Novel Paradigm for Therapeutic Intervention

The limited clinical success of the above treatment paradigms illustrates the need to investigate alternative features implicated in AD pathogenesis and exacerbation. Considerable evidence has recently emerged that chronic systemic inflammation originating in the periphery is associated with the neurodegenerative cascade in AD. Multiple systematic meta-analyses indicate elevated peripheral whole blood concentrations of inflammatory cytokines tumor necrosis factor alpha (TNF-α), interleukin 6 (IL-6), IL-1β, transforming growth factor beta (TGF-β), IL-12, and IL-18 in AD patients relative to age-matched healthy controls (Swardfager et al., 2010; King et al., 2018; Walker et al., 2019a). Increases in C-X-C motif chemokine 10 (CXCL10), a chemokine that binds to C-X-C receptor 3 (CXCR3) and subsequently primes T-cell proliferation and natural killer cell maintenance, and vascular cell adhesion protein 1 (VCAM-1), a molecule involved in microvasculature permeability, have also been observed (Lai et al., 2017). Importantly, several studies have indicated that AD patients begin presenting aberrant pro-inflammatory cytokine profiles at *early* disease stages—levels which drop precipitously with disease progression (Engelhart et al., 2004; Kuo et al., 2005). Recent investigations have reiterated these findings in whole peripheral blood and plasma samples: in a heterogenous population of patients with Lewy-body dementia (LBD), advanced AD, and MCI, significantly higher levels of IL-1β, IL-4, and IL-2 were observed in MCI patients relative to healthy controls. The severity of cognitive decline—evaluated through performance assessments including the Mini Mental State Examination (MMSE) and Addenbrooke's Cognitive Examination Revised (ACE-R)—was found to be inversely proportional to serum levels of inflammatory markers (King et al., 2018). Longitudinal clinical studies further implicate early chronic peripheral inflammation in the preponderance of neurodegenerative disease: individuals with higher levels of pro-inflammatory cytokines in midlife are at a significantly higher risk for cognitive decline as they age (Walker et al., 2019b)—those who maintained aberrant levels for multiple decades were found to be especially prone to debilitating neurodegeneration via reduced brain volume and abnormal white matter microstructural integrity (Walker et al., 2017, 2018). Altogether, these data posit a putatively temporal relationship between chronic, systemic immune activation and cognitive deterioration and suggest that inflammation—which may occur decades before the onset of AD symptoms—exacerbates or directly mediates neurodegeneration.

Integral to our current understanding of the elaborate interplay between CNS immune activity and that of the periphery is the discovery that inflammatory cytokines are capable of traversing the blood-brain barrier (BBB) (Gutierrez et al., 1993; Banks et al., 1995). Where it was once believed that the tight junctions formed by capillary endothelial lining, astrocyte sheaths, and pericytes embedded in the capillary basement membrane conferred nearly complete immune privilege (Pollack and Lund, 1990), numerous studies indicate that circulating cytokines can induce signaling in the CNS through multiple mechanisms: (1) traversal of circumventricular organs (Buller, 2001; Roth et al., 2004), (2) vagus nerve stimulation (Borovikova et al., 2000; Das, 2007), and (3) direct cytokine-endothelial interactions, which result in tight junction opening and subsequent cytokine diffusion (Walker et al., 2019a). Indeed, systemic inflammation—whether caused by infection, chronic illness, or sepsis—has been identified as the primary catalyst of BBB permeability (Le Page et al., 2018), is shown to simultaneously upregulate proinflammatory (chiefly TNF-α, IL-1β, and IL-6) and downregulate immunosuppressive (IL-1ra, IL-4, IL-10, TGF-β) markers in whole blood, serum, and plasma samples (Su et al., 2019), and activates resident microglia, which in turn locally release proinflammatory cytokines that interfere with hippocampal neurogenesis (Chesnokova et al., 2016).

The World Health Organization collectively classifies chronic inflammatory diseases as the greatest threat to human health. As of 2017, 92.1 million Americans either have doctor-diagnosed arthritis or frequently report symptoms consistent with an arthritis diagnosis—a metric predicted to increase 49% by 2040. Moreover, previous estimates, which largely rely on doctor diagnoses, drastically undervalue the prevalence of inflammatory arthritis in younger population segments: indeed, a recent study found that 1 in 3 people aged 18–64 suffer from arthritis (Jafarzadeh and Felson, 2018). The animal studies and clinical investigations reviewed herein demonstrate that conditions like rheumatoid arthritis, osteoarthritis, and osteoporosis significantly increase the risk of and putatively accelerate cognitive decline in AD-related neurodegeneration. Given their ubiquity, it is imperative to consider whether effective treatment of these peripheral disorders before AD onset can forestall or mitigate AD-related neurodegeneration and subsequent cognitive decline. Therefore, while the pathogenic mechanisms of immune dysfunction for these conditions remain elusive, a thorough review covering the pathophysiology of these disorders, identifying current treatment paradigms, and discussing evidence of comorbid associations may provide new insight into how systemic inflammation may contribute to and mediate cognitive decline.

## Inflammatory AD Comorbidities

### Rheumatoid Arthritis (RA)

#### Pathophysiology

RA is a heterogenous chronic inflammatory disease that develops as a consequence of complex interactions between the innate and adaptive immune systems and is characterized by synovial hyperplasia leading to painful joint swelling and functional impairment (Catrina et al., 2017; Ghoryani et al., 2019). Increasing evidence attributes excessive neutrophil extracellular trap (NET) formation to the stimulation and maintenance of autoimmunity and inflammation in RA (Angelotti et al., 2017). Upon initiation of the typical inflammatory cascade, neutrophils aggregate, sequester, and stimulate degradation of invading pathogens including bacteria, viruses, and some microorganisms. During a process called “NETosis,” which is thought to be elicited at least partly via local IL-8 secretion (Yipp and Kubes, 2013), neutrophils undergo “beneficial controlled suicide,” releasing a milieu of intracellular components—nucleotides, proteins, histones, and elastases—that facilitate the formation of web-like structures that bind pathogens, rendering them inert (Takei et al., 1996). In both early and late-stage RA, neutrophils demonstrate a marked proclivity toward spontaneous NET formation (Corsiero et al., 2016; Berthelot et al., 2017). Additionally, neutrophils isolated from RA patients have been shown to favor NET formation *in vitro* following antigenic and inflammatory cytokine stimulation compared to those isolated from healthy controls (Angelotti et al., 2017). Recent investigations have established that, in RA, NETs promote pathogenic interferon gamma (IFN-γ)-producing T helper subtype 1 (Th1) cell immune responses by increasing secretion of dendritic cell costimulatory molecules cluster of differentiation 80 (CD80) and CD86, as well as pro-inflammatory cytokine IL-6 (Papadaki et al., 2016). Another pathological mechanism that has recently gained traction is cyclical NETosis-mediated autoantibody production. RA neutrophils strongly express protein-arginine deiminase 4 (PAD4), an enzyme that catalyzes the conversion of specific arginine residues to citrulline, and as a result manufacture citrullinated forms of fibrinogen and histones H2A and H2B (Berthelot et al., 2017). These proteins, in turn, are recognized by antibodies to citrullinated protein antigens (ACPAs) which incite an autoimmune response that prompts inflammatory cytokine secretion, further neutrophil infiltration, and NET formation (Yipp and Kubes, 2013). Thus, NET-produced citrullinated proteins fuel the ACPA autoimmune response within the RA synovium, producing a positive-feedback loop that stimulates exponential immune activity.

**Figure 1:**
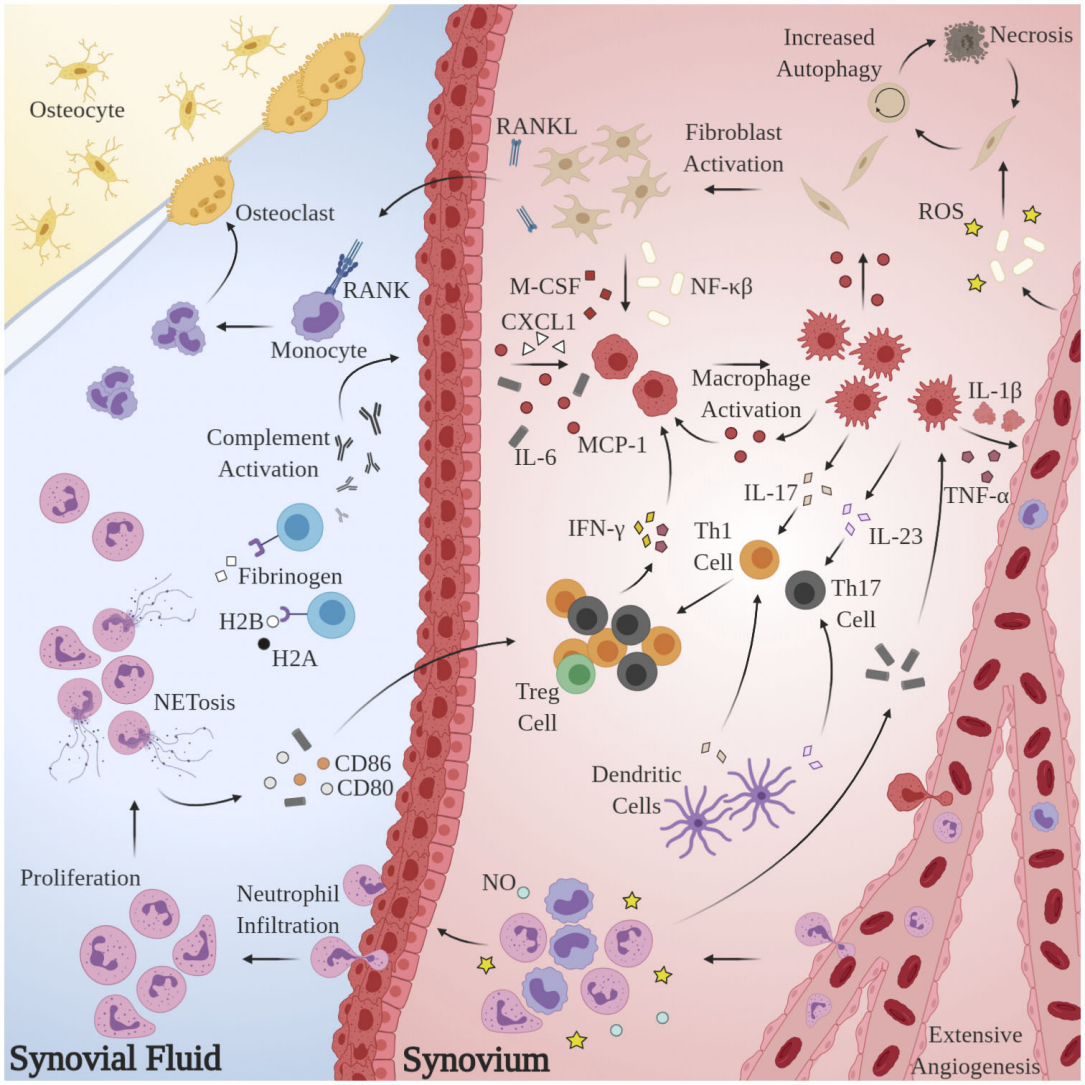
Model for the generation and maintenance of chronic inflammation in RA. Pathological neutrophils manufacture citrullinated forms of fibrinogen and histones H2A and H2B which are recognized by proximal APCAs, prompting activation of the complement cascade and secretion of chemoattractant and pro-inflammatory cytokines CXCL1, IL-6, and MCP-1. Local macrophages are “activated” by this milieu, inciting extensive angiogenesis, activation, and proliferation of T-lymphocytes and synovial fibroblasts and further recruitment of circulating macrophages, monocytes, and neutrophils. Pro-inflammatory Th subsets dominate, owing largely to the byproducts of NETosis. Elevated RANKL production and osteoclast proliferation have also been observed.

Substantial research has implicated aberrant pro-inflammatory macrophage activity in the pathogenesis and maintenance of synovitis in RA. TNF-α is heavily upregulated in the synovial fluid (SF) of RA patients (Chu et al., 1991), is known to directly impair endothelial function by inciting production of nuclear factor κB (NF-κB) and reactive oxygen species (ROS) (Di Minno et al., 2015), and plays a pivotal role in disease pathogenesis (Ursini et al., 2017). Immunohistological assessments of excised synovial tissues reveal that macrophages are the principal TNF-producing cells in the inflamed RA joint (Udalova et al., 2016). Localized abundance of TNF-α and macrophage secretion of IL-8 and monocyte chemoattractant protein 1 (MCP-1) results in the recruitment of peripheral monocytes and neutrophils and activation of synovial fibroblasts, which perpetuate the inflammatory response via addition of IL-1β (Hamilton et al., 1993; Shigeyama et al., 2000). Activated synovial fibroblasts, in turn, produce receptor activator of nuclear factor κB ligand (RANKL) and macrophage colony stimulating factor (M-CSF) which elicit osteoclast proliferation and enforce pro-inflammatory macrophage polarization, respectively (Braun and Zwerina, 2011). The pathological macrophage secretome is likewise implicated in the dysregulation of adaptive immune processes: IL-23 stimulates the activation and proliferation of Th17 cells, putative regulators of autoimmunity in RA (Miossec and Kolls, 2012). Sustained macrophage IL-12 expression has been shown to upregulate Th1 activity (Aarvak et al., 1999). Indeed, the maintenance of the pro-inflammatory environment in RA appears to be due, at least in part, to the disruption of Th(1,17)/Treg balance (Wang W. et al., 2012), but further investigation is required to delineate the source(s) of this phenotypic shift. Figure 1 summarizes these mechanisms.

#### Advancements in Current Treatments

The central role of TNF-α in synovial hyperplasia is emphasized by the clinical benefits conferred by anti-TNF drugs. While the therapeutic efficacy of conventional synthetic and biologic disease-modifying antirheumatic drugs (DMARDs) are thoroughly discussed elsewhere (Aletaha and Smolen, 2018), of particular note are antibody-based TNF antagonists Etanercept, Golimumab, and Certolizumab pegol. In a randomized, controlled clinical trial involving 234 patients with active RA, twice-weekly subcutaneous injections of Etanercept significantly reduced disease activity in a dose-dependent fashion (Moreland et al., 1999). In patients that had discontinued use of conventional TNF-α inhibitors due to lack of effectiveness, Golimumab significantly improved multiple patient outcomes, including swollen joint count, tender joint count, and patient assessments of pain, throughout the entire 24-week trial period (Smolen et al., 2009). Its increased effectiveness is attributed to its entirely human architecture and ability to bind both soluble and transmembrane TNF (Radner and Aletaha, 2015). Comparable therapeutic efficacy was observed in DMARD inadequate responders with Certolizumab pegol which, significantly, required half the administration frequency relative to standard DMARDs (Fleischmann et al., 2009).

Recent preclinical studies have explored more targeted approaches for correcting deviant mechanisms within the innate and adaptive immune systems in RA. As mentioned above, M-CSF is heavily upregulated in the SF of RA patients. Binding of the cytokine to its cognate receptor CSF1R is required for osteoclastogenesis and TNF-α induced osteolysis. Prophylactic administration of muAB5, a CSF1R antagonist, significantly reduced production of IL-6, CXCXL8, C-C motif chemokine ligand 2 (CCL2), CCL7, and matrix metalloproteinase 9 (MMP-9) in a collagen-induced arthritis (CIA) mouse model of RA (Garcia et al., 2016). Yin yang 1 (YY1) is a transcription factor that regulates multiple complex biological functions, has recently gained attention as a mediator of autoimmune disease, and is over-expressed in both RA patients and CIA mice. Lentiviral YY1 deactivation attenuated IL-6 production, reduced Th17 activity, and slowed disease progression in CIA mice (Lin et al., 2017). Jiang et al. targeted synovial angiogenesis and discovered that subcutaneous IL-35 administration attenuated arthritis in CIA mice via Th17 suppression, Treg stimulation, and inhibition of VEGF-mediated angiogenesis (Jiang et al., 2016).

#### Relation to AD

Numerous preclinical, systematic, and meta-analysis studies have implicated RA in the pathogenesis of LOAD. Raised whole blood and serum levels of several pro-inflammatory cytokines and adaptive immune players (chiefly TNF-α, IL-1β, IL-6, IFN-γ, Th1, and Th17) have been extensively studied for their involvement in the pathogenesis of both RA and AD (Ravaglia et al., 2007; Aletaha et al., 2008; Trollor et al., 2012; Pope and Shahrara, 2013; Schoels et al., 2013; Chi et al., 2017). A recent study found that inducing arthritis in APP/PS1 mice—the canonical murine model of AD—led to glial activation and exacerbation of amyloid pathology (Kyrkanides et al., 2011). Perhaps more compellingly, a nationwide cohort study found that the incidence of AD and other dementia-related illnesses is higher in RA patients than that of the general population (Lin et al., 2018). An independent nested case-control study of more than 8.5 million adults validated this disparity and found that it was maintained in both the young (average age 42.1 years) and the elderly (aged 65+) (Chou et al., 2016). In fact, the presence of any inflammatory joint disorder (OR: 1.96), but especially RA (OR: 2.77) is significantly associated with AD-related cognitive decline later in life—a correlation that remains significant when considering AD only and not general dementia (2.49) (Wallin et al., 2012). Interestingly, Vitturi et al. reported RA patients demonstrate evidence of cognitive impairment independent of canonical AD mechanisms earlier in life: RA patients scored significantly lower in MMSE and MoCA cognitive performance assessments relative to healthy controls (*p* < 0.001). General neuropsychiatric impairment was also found to be more prevalent in RA patients (59.5%) than in age-matched controls (17.1%; *p* < 0.001) (Vitturi et al., 2019). A recent systematic review recapitulated these findings and found that RA patients—predominantly women—exhibit significantly lower scores in attention, concentration, memory, and verbal function than age-matched controls. Finally, treatments including DMARD methotrexate (Judge et al., 2017) and prescription NSAIDs (Zandi and Breitner, 2001; Weaver and Carter, 2008), which target inflammation remission in RA patients, have been found to decrease risk of AD-related dementia particularly when administered early in disease. These data posit a putative, temporal relationship between chronic inflammation in RA and the onset and exacerbation of cognitive impairment in AD. Moreover, they highlight the importance of implementing treatments before AD symptom onset that target aging-associated systemic inflammation.

### Osteoarthritis (OA)

#### Pathophysiology

OA is a progressive chronic inflammatory disease identified via gradual deterioration and loss of articular cartilage with concomitant structural and functional changes throughout the joint, including the synovium, meniscus, periarticular ligaments, and subchondral bone (Buckwalter and Mankin, 1998; Mobasheri and Batt, 2016). While chronic immune activation in OA is considered low-grade relative to RA (Robinson et al., 2016), synovial explants and synovial fluid extracted from OA patients consistently demonstrate elevated levels of pro-inflammatory mediators TNF-α, IL-1β, IL-6, IL-8, IL-15, IL-17, IL-18, IL-21, PGE2, NO, and various complement components implicated in perpetuating immune activation (Blom et al., 2007; Robinson et al., 2016; Krishnasamy et al., 2018; Mora et al., 2018; Griffin and Scanzello, 2019). Unlike RA, the clinical manifestations of OA (pain, joint range of motion, radiographic pathology) are heterogenous; nevertheless, canonical features of inflammatory arthritis, including perivascular fibrosis and lymphoid follicles, are essentially conserved (Griffin and Scanzello, 2019). Moreover, like RA, synovial macrophages are considered key mediators of synovitis, synovial hyperplasia, osteophytosis, and inflammatory factor secretion (Blom et al., 2007; Bondeson et al., 2010). The number and concentration of macrophages is significantly upregulated in synovial tissue of OA patients and is known to be proportional to the severity of articular cartilage degradation (Kraus et al., 2016). Recent investigations posit a central role for macrophages in established destructive pathways: upon activation by damage-associated molecular patterns (DAMPs; the byproducts of cartilage degeneration) (Roh and Sohn, 2018), complement membrane attack complexes (MACs) (Ricklin et al., 2016), and pathological chondrocytes and synovial fibroblasts via toll-like receptors (TLRs) and CCR2, respectively, macrophages secrete IL-1, which induces activation and proliferation of matrix-metalloproteases MMP1, MMP3, and MMP13, as well as PGE2 (Bondeson et al., 2010; Griffin and Scanzello, 2019), cardinal mediators of cartilage catabolism. Various *in vivo* studies incriminate macrophages further: Blom et al. observed that macrophage depletion prior to OA induction via intra-articular administration of clodronate significantly decreased MMP-mediated cartilage damage in a CIA murine model (Blom et al., 2007). More recently, it was found that selective inhibition of macrophage pyroptosis—a novel apoptotic pathway implicated in OA (Vande Walle and Lamkanfi, 2016)—rescued synovial fibrosis and reduced inflammatory factor expression (Zhang L. et al., 2019).

The adaptive immune constituents in the OA synovium share many of the pathological features exhibited in inflammatory arthritis. CD3^+^ T cells dominate synovial infiltrates, and CD4^+^/CD8^+^ cells propagate at levels comparable to those seen in RA synovial explants (Haseeb and Haqqi, 2013). Pro-inflammatory Th1 T cell subsets dominate their largely immunosuppressive (Th2) counterparts (Li et al., 2017) and directly contribute to the upregulation of inflammatory cytokines IL-2 and IFN-γ found in most OA patients. While no conclusive data exist identifying putative antigens responsible for autoantibody production, CD20^+^ B-lymphocytes are found in significantly higher concentrations in sclerotic regions of subchondral bone (Weber et al., 2019a) and recent clonal analyses indicate OA B-cells undergo antigen driven activation suggestive of clonal selection (Da et al., 2007; Zhu et al., 2020).

**Figure 2:**
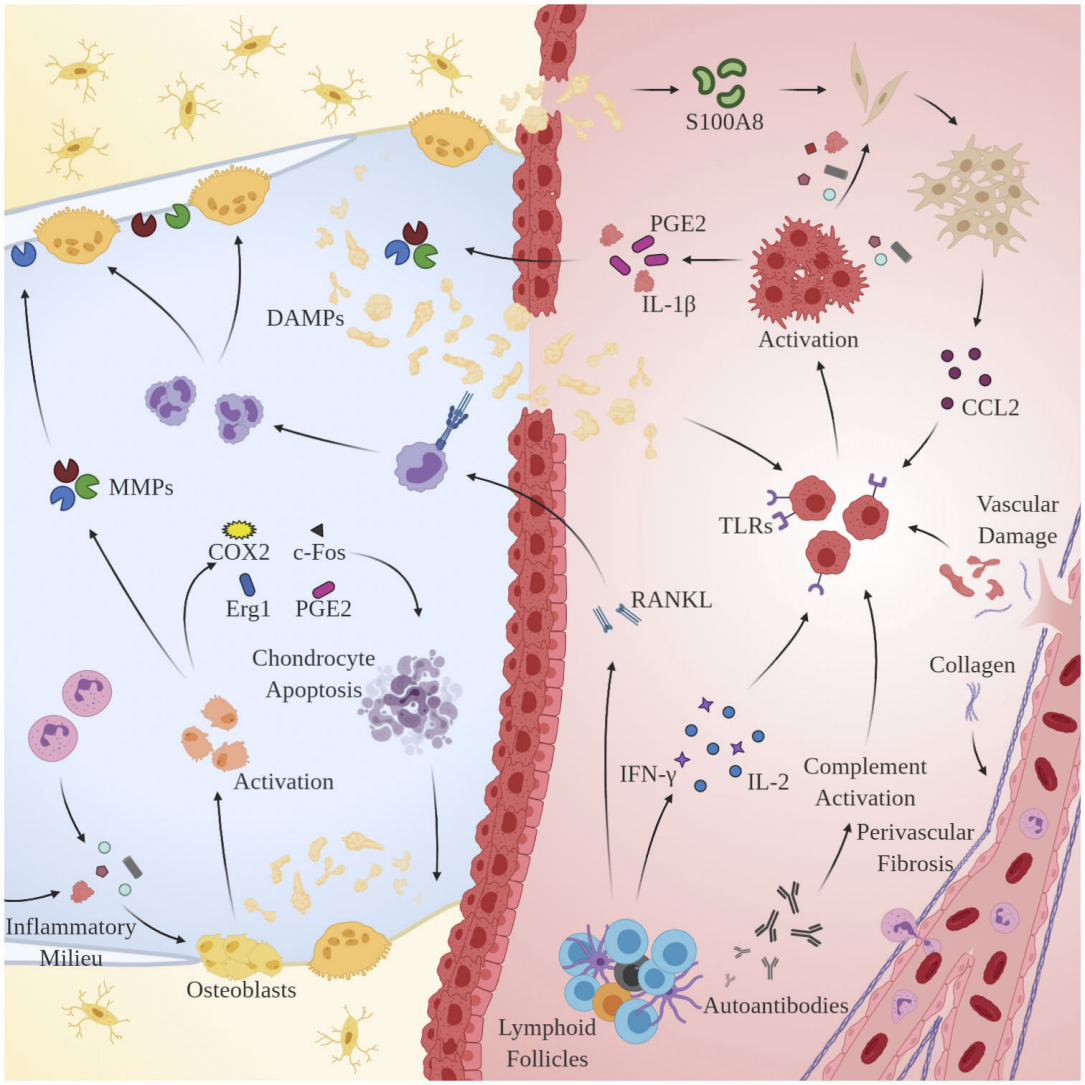
Mechanisms of chronic immune hyperactivity in OA. Clinical features of inflammatory arthritis, including perivascular fibrosis and lymphoid follicles, are conserved. Upon activation by DAMPs, MACs, chondrocytes and synovial fibroblasts via TLRs and CCR2, respectively, macrophages secrete IL-1β, inducing proliferation of MMPs 1, 3, and 13, and PGE2 production. Th1 cells dominate and directly contribute to upregulation of inflammatory cytokines IL-2 and IFN-γ. B-cells undergo clonal selection and are implicated in RANKL production.

Remarkably, osteoblasts have garnered increased attention as another key player in OA pathogenesis. Alterations in the physicochemical environment of subchondral bone may be linked to the progression of OA, as osteoblast phenotype is known to be modulated by a variety of stimuli including intraosseous pressure, fluid shear, mechanical loading, and local oxygen saturation (Hillsley and Frangos, 1994; Dodd et al., 1999; Warren et al., 2001). Indeed, preclinical studies in guinea pig models of OA have demonstrated that venous outlet syndrome and decreased perfusion directly precede and radiographically coincide with bone resorption and cartilage degeneration (Imhof et al., 1997; Watt, 2009). Additionally, Tanaka et al. observed that osteoblasts respond to changes in strain-induced fluid flow by synthesizing cytokines involved in the extracellular matrix (ECM) changes observed in OA including transcription factors c-Fos and Egr1, intracellular inflammatory mediators COX2, PGE2, and NO, and catabolic enzymes MMP1, MMP3, and MMP13 (Tanaka et al., 2005). Likely as a result of interactions with DAMPs and sustained exposure to the pro-inflammatory microenvironment observed in OA, osteoblasts undergo a discernable, pathological phenotypic shift that accelerates disease progression by interacting with key regulators of cartilage homeostasis: synovial chondrocytes. Conditioned media taken from OA-derived osteoblasts has been shown to enhance GAG release from normal cartilage (Westacott et al., 1997). Further, co-cultures of OA-derived osteoblasts and chondrocytes result in reduced expression of COL2A1, aggrecan, PTHrP/PTH-R, and SOX9 (Sanchez et al., 2005b), increased expression of OSF-1, MMP3, and MMP13 (Sanchez et al., 2005a), and induction of chondrocyte hypertrophy and matrix calcification via p38 and ERK-1/2 suppression (Aaron et al., 2017). See Figure 2 for an overview of the pertinent pathological mechanisms.

#### Advancements in Current Treatments

Current clinical therapeutic goals for OA include inflammation and concomitant pain remission, ameliorating existing damage to or stimulating regeneration in articular cartilage, maximizing range of motion, and in general enhancing patient quality of life. Of the currently available treatments, topically and orally administered NSAIDs still represent the most prescribed medications for managing OA-related pain (Mora et al., 2018; Nakata et al., 2018), though other treatments paradigms have demonstrated some clinical success. Duloxetine, a serotonin and norepinephrine reuptake inhibitor originally prescribed for severe depression disorders, performed better than placebo at reducing pain and improving function when administered for at least 10 weeks (Citrome and Weiss-Citrome, 2012; Wang Z. Y. et al., 2015). Correction of dysfunctional pain pathways is considered the primary mechanism of action. Corticoids, another common therapy modality, exert anti-inflammatory effects by acting directly on nuclear receptors, decreasing production of IL-1, leukotrienes, prostaglandins (PGs), and MMPs (Levy et al., 2018). Significantly, >50 mg doses of prednisone (an NF-κB inhibitor) have been shown in multiple clinical trials to confer more lasting pain relief compared to other corticoid-based therapies (Bellamy et al., 2006; Law et al., 2015; Buyuk et al., 2017); however, a recent meta-analysis suggests that longitudinal corticoid administration may contribute to cartilage loss and degenerative OA pathology, suggesting that systemic anti-inflammatories possess limited efficacy in chronic conditions (Zeng et al., 2019).

Hyaluronic acid (HA)—a glycosaminoglycan that provides viscous lubrication and shock-absorbing properties in healthy synovial tissue—is a common intraarticular supplement for the management of mild to severe OA (Altman et al., 2016). While some studies purport exogenous HA enhances endogenous HA and proteoglycan synthesis, promotes articular cartilage regeneration, and inhibits synovial production of pro-inflammatory cytokines (Migliore and Procopio, 2015), evidence for long-term clinical efficacy is conflicting (Richards et al., 2016; Altman et al., 2018; Pelletier et al., 2018) and the American Academy of Orthopedic Surgeons no longer recommends IA HA injection for clinical use (Jevsevar, 2013). Platelet-rich plasma—whole blood fractions prepared via centrifugation of autologous blood—may provide a viable alternative. Multiple favorable patient outcomes were observed in a series of randomized controlled trials (Cerza et al., 2012; Patel et al., 2013; Vaquerizo et al., 2013) due largely to its regenerative effect and anti-inflammatory potential (Shen et al., 2017).

#### Relation to AD

Systemic chronic inflammation has been implicated in the initiation and cyclical aggravation of a variety of age-related disorders including OA and AD (Weber et al., 2019b). Systematic reviews have identified multiple potential mechanisms through which the chronic low-grade inflammation in OA may contribute to AD-related neurodegeneration: (1) disruption of the BBB and subsequent influx of peripheral pro-inflammatory cytokines, (2) active transport of pro-inflammatory cytokines across the BBB, (3) a chain of activation events including brain endothelial cells, perivascular cells, and brain parenchymal cells, and (4) aberrant peripheral nervous system (PNS) activity (e.g., communications between peritoneal cavity and neuronal populations in the brain stem) (Banks et al., 2002; Konsman et al., 2002). Various studies have demonstrated significant overlap between the pathological cytokine profiles observed in OA and AD: one such work found that high-mobility group box 1 (HMGB1) and its cognate receptor for advanced glycation end products (RAGE) are found at greatly elevated levels in both OA (Sun et al., 2016) and AD (Festoff et al., 2016). Multiple animal studies have linked OA to AD exacerbation and pathogenesis: induction of OA in APP/PS1 mice resulted in accelerated development of Aβ plaques and greater plaque deposition at later timepoints compared to OA^−^ controls (Kyrkanides et al., 2011). Moreover, transgenic mice expressing the human APOE ϵ4 allele—an allele associated with greater risk of LOAD onset—exhibited significantly greater synovial thickening and 32% more cartilage damage relative to APOE ϵ3 mice following 42 days of OA induction (de Munter et al., 2017). This suggests that patients carrying the pathological ϵ4 allele may be more susceptible to OA and other peripheral inflammatory diseases in addition to AD.

The results of epidemiological and longitudinal meta-analyses paint a similar picture. Age- and gender-adjusted cohorts of OA patients were found to be at a significantly greater risk for developing dementia later in life (OR: 1.36, *p* < 0.0001) (Weber et al., 2019b). A recent nationwide cohort study in Taiwan established a similar correlation, finding that OA patients were 25% more likely to have dementia (Adjusted HR: 1.25, *p* < 0.001) (Huang et al., 2015). In a study including 21,982 Appalachian adults aged 40 and older, participants with OA were found to be 80% more likely to report frequent memory loss independent of sleep or mood disorders (OR: 1.8, *p* < 0.001) (Innes and Sambamoorthi, 2018). Interestingly, in an investigation representing nearly 42.7 million Americans aged 65 or older, patient-reported pain, and the extent to which pain interfered with activities of daily living, was found to be significantly and positively correlated with the incidence of AD and related dementias, both in the presence (OR: 1.37) and absence (OR: 1.44) of OA (*p* < 0.005) (Ikram et al., 2019). Further investigation is required to decouple the contributions of pain to disease pathology.

### Osteoporosis (OP)

#### Pathophysiology

**Figure 3:**
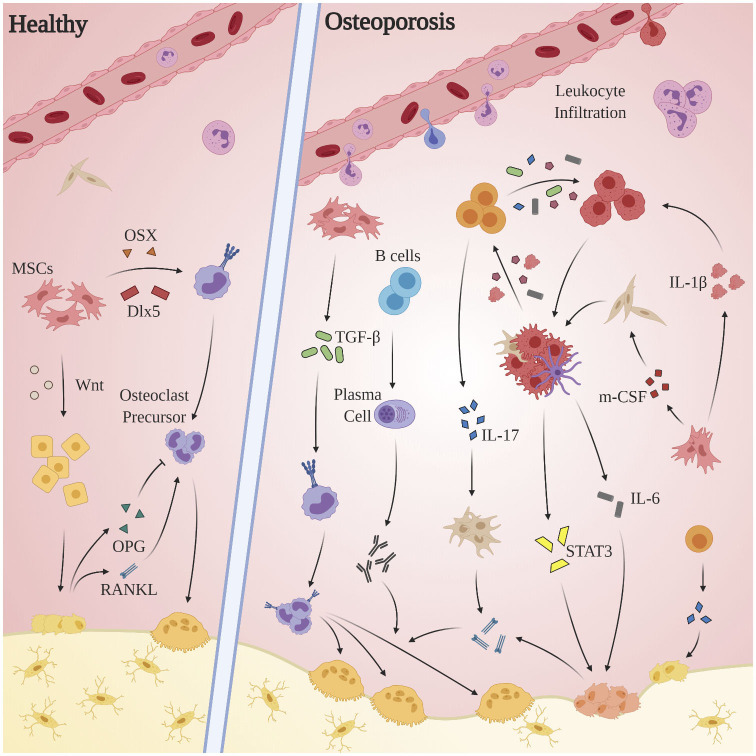
Aberrant immune mechanisms in OP. Chronic pathological levels of pro-inflammatory cytokines and mediators promote bone resorption via osteoclast differentiation and activation, enhancement of RANKL expression, and the inhibition of osteoblast survival. The inflammatory milieu produced by macrophages, dendritic cells, and local fibroblasts incites proliferation of Th17 cells, which in turn express RANKL, promote upregulation of RANKL expression by osteoblasts and fibroblasts, and exacerbate the M1 polarization of macrophages. Multiple players contribute to osteoclastogenesis—both indirectly, as through activated stromal cell secretion of RANKL, and directly as by promoting osteoclast differentiation through activation of TGF-β. The STAT3 and CXCL1/R1 axes, while clearly of clinical significance, remain obfuscated and merit further investigation.

OP is an age-related bone disorder characterized by reduction in bone mass and impairment of microarchitecture resulting in fragility fractures and a preponderance in activity of osteoclasts over osteoblasts (Pietschmann et al., 2016). Pathological bone resorption in OP is caused in part by changes in relative concentrations of RANKL and osteoprotegerin (OPG): RANKL is a type II transmembrane protein expressed by osteoblasts, proximal T-lymphocytes, and bone marrow stromal cells. Binding of RANKL to its cognate receptor RANK induces terminal differentiation of preosteoclasts and subsequent bone resorption. OPG, produced by osteoblasts (Hofbauer et al., 1999) and select B-cells, acts as a competitive inhibitor for RANKL (Awasthi et al., 2018). Under normal circumstances, completion of bone resorption initiates bone formation via recruitment of preosteoblast cells, during which factors including TGF-β, IGF-1, IGF-2, BMP-2, PDGF, and FGF inform differentiation of mesenchymal stem cells into osteoblasts (Clarke, 2008). Alternatively in OP, chronic pathological levels of pro-inflammatory cytokines and mediators promote bone resorption via osteoclast differentiation and activation, enhancement of RANKL expression, and the inhibition osteoblast survival (Clowes et al., 2005). Indeed, systemic inflammation is implicated in the dysregulation of multiple processes related to bone homeostasis: OP pathology propagates through a complex interplay of endocrine (estrogen; Almeida et al., 2017; Levin et al., 2018; Wu et al., 2018; Farr et al., 2019, parathyroid hormone; Camirand et al., 2016; Noordin and Glowacki, 2016; Williams et al., 2018; Lou et al., 2019, androgen; Shin et al., 2018; Joseph et al., 2019) immune (T-lymphocytes, cytokines), small molecule (Vitamin D; Ebeling and Eisman, 2018; Shill et al., 2019), and canonical signal pathway (Wnt/β-catenin; Johnson and Recker, 2017; Amjadi-Moheb and Akhavan-Niaki, 2019) regulators (see Figure 3).

Inflammatory cytokine-mediated bone resorption appears to occur through a variety of mechanisms. Both T- and B-lymphocytes have been shown to constitutively overexpress RANKL in pro-inflammatory conditions (Pietschmann et al., 2016; Srivastava et al., 2018). The inflammatory milieu produced by macrophages, dendritic cells, and local fibroblasts (TGF-β, IL-6, IL-1β, IL-23) incites proliferation of Th17 cells, which in turn promote bone resorption via RANKL expression (Dar et al., 2018c), upregulation of RANKL expression by osteoblasts and fibroblasts via IL-17 (Raphael et al., 2015), and exacerbation of the pro-inflammatory polarization of macrophages through secretion of IL-6, IL-17, TNF-α, and IFN-γ (Komatsu and Takayanagi, 2012). Activated B-lymphocytes, in addition to secreting TNF-α (Weitzmann, 2014), generates autoantibodies implicated in accelerating osteoclastogenesis (Pietschmann et al., 2016). TNF-α secreted by dendritic cells, macrophages, and CD4^+^/CD8^+^ cells is purported to act both indirectly by activating stromal cell secretion of RANKL, M-CSF, and IL-1, and directly by promoting osteoclast differentiation through activation of TGF-β (Al-Daghri et al., 2017). While data concerning inflammatory cytokine expression profiles in OP patients is limited, one study of over 100 post-menopausal OP patients found elevated pro-inflammatory cytokine levels (TNF-α, IL-1β, IL-6) were inversely correlated with expression of markers involved in inflammation remission (IL-4) and osteogenesis (osteocalcin) (Al-Daghri et al., 2017). STAT3 and the CXC (L1/R1) axis also merit further investigation: in an RA-induced murine population, STAT3 activation driven by pro-inflammatory cytokine expression led to increased RANKL-mediated bone loss, and STAT3 inhibition via cycloheximide significantly reduced expression of IL-6 family cytokines and RANKL (Mori et al., 2011). In a study comparing pre- and post-menopausal healthy controls to post-menopausal OP patients, CXCL1 concentrations were inversely correlated to bone mineral density and were directly proportional to bone turnover (TRACP-5b, NTx) and inflammatory (IL-1β, IL-6) markers (Chen et al., 2016), suggesting CXCL1 may be correlated to degree of OP development. Viral-mediated suppression of CXCR1 transcription resulted in a distinct reduction in RANKL-induced osteoclastogenesis (Wojdasiewicz et al., 2019).

#### Advancement in Current Treatments

Antiresorptive drugs are the most common therapy for osteoporotic patients and include selective estrogen response modulators (SERMs), bisphosphonates, and antibody-based RANKL inhibitors (Chapurlat and Genant, 2015). Estrogen is known to mediate bone turnover by directing calcium and Vitamin D homeostasis and conditionally promoting upregulation of cytokines that either incite or inhibit bone resorption (Lizneva et al., 2018). That OP is overwhelmingly presented by post-menopausal women (four times more common in women over 50 than similarly aged men) further solidifies the preponderance of estrogen in disease pathology. Thus, until recently, exogenous estrogen administration was a popular antiresorptive therapy: several controlled trials have demonstrated its ability to prevent bone mineral density (BMD) loss and reduce the risk of hip fractures by ~30% (Chapurlat and Genant, 2015). Unfortunately, bone loss resumes at post-menopausal levels following cessation of therapy (Greendale et al., 2002) and prolonged treatment is linked to aberrant blood coagulant activity and significant breast cancer risk (Rossouw et al., 2009; Gennari et al., 2016).

The therapeutic mechanisms and longitudinal efficacies of SERMs, bisphosphonates, and RANKL inhibitors have been extensively reviewed elsewhere (Gennari et al., 2016). Moreover, the limited clinical potency and risk factors associated with existing treatments underscores the need to target alternative pathways contributing to disease pathology. Recent investigations have identified multiple treatments that remediate bone loss through modulating immune activity. B cell depletion via rituximab reduced synovial RANKL, expression of RANK^+^ osteoblasts, and sera levels of bone turnover markers in patients with inflammatory OP (Wheater et al., 2011; Boumans et al., 2012). In ovariectomized and post-menopausal murine models of OP, administration of D-mannose (Liu et al., 2020), *Bacillus calusii* (Dar et al., 2018a), and *Lactobacillus acidophilus* (Dar et al., 2018b) attenuated bone loss, reduced expression of pro-inflammatory cytokines IL-6, IL-17, TNF-α, and IFN-γ, and increased expression of anti-osteoclastogenic factor IL-10 by stimulating the proliferation of Treg cells, restoring Treg/Th17 balance. Antibody-based TNF-α inhibition in a rat model of OP elevated bone density, simultaneously increased and decreased OPG and RANKL expression, respectively, and enhanced osteogenic differentiation of endogenous stromal cells (Yu et al., 2019). Collectively, these and other studies purport inflammation as a viable target for therapeutic intervention in OP.

#### Relation to AD

While AD and OP initially appear pathologically and immunologically distinct, the results of numerous studies suggest a bidirectional and mutually antagonistic interplay between the two age-related disorders. Patients with AD exhibit, on average, significantly reduced hip BMD and retain a nearly 2-fold risk of hip fracture relative to healthy age-matched controls (Chen and Lo, 2017). Elevated TNF-α levels observed in AD patients, even before the onset of cognitive impairment, are known to induce osteoclastogenesis, inhibit bone formation by suppressing Wnt signaling, and accelerate cartilage destruction via production of MMPs and disintegrin and metalloproteinase with thrombospondin motifs (ADAMTSs) (Osta et al., 2014). Bone turnover proteins osteocalcin (OCN), osteopontin (OPN), and sclerostin have been shown to exert potent neurological effects *in vivo* (Yuan et al., 2019a): OCN can traverse the BBB, enhance synthesis of serotonin, dopamine, and noradrenaline, inhibit GABA secretion, and bind to neurons in the brainstem, midbrain, and hippocampus (Oury et al., 2013). In a recent study, intravenous (IV) injection of plasma derived from OCN^+/+^ 3 month-old mice rescued cognitive function of 16 month-old WT mice, but this therapeutic effect could not be replicated with OCN^−/−^ mice, suggesting OCN may play a role in mitigating age-related cognitive deficits (Khrimian et al., 2017). OPN is found in higher levels in the plasma of AD patients (Comi et al., 2010; Carecchio and Comi, 2011), is known to enhance bone resorption (Luukkonen et al., 2019), and reduces Aβ burden in murine models of AD (Rentsendorj et al., 2018). Moreover, pathological variants of Aβ propagate in osteoporotic bone (Xia et al., 2013) and BMD has been shown to be inversely correlated with Aβ and APP expression in vertebral trabecular bone specimens (*r* = −0.617 and −0.531 for Aβ_42_ and APP, respectively) (Li et al., 2014). *In vitro*, Aβ_42_ enhances RANKL-induced bone resorption by silencing inhibitor of nuclear factor kappa B (IκB), subsequently enhancing NF-κB activity (Li et al., 2016). Finally, in AD, microglia-like cells originating from bone marrow traverse the BBB and migrate into the brain in a chemokine-dependent manner (El Khoury and Luster, 2008), giving further credence to the hypothesis that peripheral inflammation can directly contribute to pathological microgliosis and subsequent neuronal degradation.

## Additional Mechanisms Underlying Systemic Pathology

While no single theory exists concerning the underlying mechanisms of these systemic disorders, cellular senescence, dysregulation in peripheral nervous system activity, and a disruption in autophagic homeostasis are considered hallmark artifacts of arguably the most significant risk factor for all the conditions discussed above: aging. Cellular and immuno-senescence are highly conserved features in age-related inflammatory bone and joint disorders, as well as in CNS diseases including LOAD (Rodier and Campisi, 2011; Muñoz-Espín and Serrano, 2014). Cell cycle suppression genes are heavily upregulated with age and are implicated in the generation and maintenance of chronic systemic inflammation and exacerbation of disease pathology (Lebrasseur et al., 2015). Aberrant peripheral nervous system activity provides a physiological basis for the intimate, bidirectional relationship between bone and joint disorders and dysregulated neurological homeostasis. Not only are autonomic structures among the first impacted in neurodegenerative illness, but select neuropeptides highly expressed in bone are known to drive a host of osteo-homeostatic processes including bone reformation, hematopoietic progenitor cell (HPSC) niche maintenance, and innate and adaptive immune activity (Asada et al., 2013). Finally, global reduction in autophagy has been reported to exacerbate age-associated inflammation and accelerate the progression of degenerative diseases (Cuervo and Dice, 2000). Conversely, the maintenance of proper autophagic activity has been credited with heightened longevity and resistance to a host of age-related conditions (Arensman and Eng, 2018). Therefore, while complex disorders like AD result from a wide range of multifactorial mechanisms, a comprehensive understanding of these age-related phenomena is crucial to the development of effective interventional therapies.

### Age-Related Cellular/Immuno-Senescence

Tissues affected in a variety of age-related diseases exhibit a preponderance of senescent cells characterized by cell cycle arrest, apoptosis resistance, and chronic secretion of preferentially pro-inflammatory molecules (Rodier and Campisi, 2011; Muñoz-Espín and Serrano, 2014). In conventional aging, cells assume a senescence-associated secretory phenotype (SASP) which is believed to contribute to age-related tissue inflammation (Lebrasseur et al., 2015). While SASPs surface from a variety of factors, including cell type, mechanism of senescence induction, and hormonal milieu (Wiley et al., 2016), inflammatory cytokines, including IL-6 and IL-8, are highly conserved in SASPs and are believed to play a crucial role in maintaining SASP signaling and senescence (Acosta et al., 2008; Coppé et al., 2008; Lasry and Ben-Neriah, 2015).

In AD, aggregating Aβ_1−42_ peptides have been shown to directly trigger expression of senescence-associated marker β-galactosidase in oligodendrocyte precursor cells (OPCs) (Osso and Chan, 2019), indicating that senescent OPCs commonly observed in AD brains may be generated through direct stress from Aβ aggregates rather than, or in addition to, canonical replicative senescence mechanisms. Indeed, in 5XFAD murine models of AD, the expression of cell cycle suppression genes *p53, p16*, and *p21* were all upregulated in hippocampal homogenates. *P16* demonstrated the most significant discrepancy, increasing over 5-fold following the onset of pathology—a result consistent for both genomic and proteomic characterizations. Importantly, *p16* expression was inversely correlated with cognitive performance and immunofluorescent staining revealed predominantly neuronal localization of the gene. These results were recapitulated *in vitro*: the administration of 10 μM oligomeric Aβ significantly upregulated *p16* (but not *p53*) in neuron monoculture (Wei et al., 2016). A similar senescent phenotype was discovered in aged APP/PS1ΔE9 mice and both short- and long-term administration of quercitin, a senolytic drug providing targeted ablation of senescent cells, conferred multiple benefits. Short-term treatment eliminated senescent OPCs, ramified pathological microglia proximal to plaques, and reduced IL-6 levels. Long-term therapeutic intervention before the onset of plaque pathology reduced overall hippocampal plaque burden later in disease and attenuated overall inflammatory cytokine secretion (Zhang P. et al., 2019).

*P16* and *p21* are also known to be heavily upregulated in OP bone (Farr et al., 2019). In highly enriched cell populations derived from murine bone and bone marrow with no *in vitro* culture, expression of *p16* was greater in B- and T-lymphocytes, myeloid cells, osteoblast progenitors, osteoblasts, and osteocytes in 24 month-old mice relative to 6 month-old mice. Conventional aging was attributed to an over 500% increase in senescent osteocytes (Khosla et al., 2018). Indeed, as they age, bone marrow derived MSCs are known to not only lose their functional and regenerative capabilities, but also develop an increased propensity toward replicative senescence, contributing to chronic systemic inflammation and exacerbation of disease pathology (Qadir et al., 2020). Targeting senescent cells using genetic (Baker et al., 2011), senolytic (Yi et al., 2016), or inflammatory SASP-inhibiting compounds (Xu et al., 2015) for 2–4 months markedly improved bone mass and microarchitecture in trabecular and cortical bone.

### Aberrant Peripheral Nervous System (PNS) Activity

Autonomic dysfunction—postulated to surface due to deficits in cholinergic function—is common in patients with dementia (Allan et al., 2007; Femminella et al., 2014). In an observational study, MCI patients were found to be ~5.6 times more likely to demonstrate parasympathetic dysfunction than age-matched healthy controls (Collins et al., 2012). Central autonomic structures, including the hypothalamus, amygdala insula, and locus coeruleus are among the first neural structures afflicted in neurodegenerative illnesses like AD (Ahmed et al., 2015). These alterations culminate in markedly changed neuropeptide levels in the brains and cerebrospinal fluid (CSF) of AD patients—among them, reduced cortical calcitonin gene-related peptide (GCRP) (Choi et al., 2014) and vasoactive intestinal peptide (VIP) (Zhou et al., 1995; Sterniczuk et al., 2010), diminished CSF Substance P (SP) (Friedberg et al., 1991; Quigley and Kowall, 1991; Waters and Davis, 1997), and elevated norepinephrine (NE) (Gannon and Wang, 2019), tyrosine hydroxylase (TH) (Szot et al., 2006, 2007), dopamine β hydroxylase (DBH) (Giubilei et al., 2004), and neuropeptide Y (NPY) (Allen et al., 1984).

Importantly, these and other neuronal products are expressed in bone and have been shown to exert multiple immunomodulatory and osteo-homeostatic pathological deviations in the periphery (Asmus et al., 2000). CGRP increases proliferation and reduces apoptosis of osteoblast progenitors, enhances osteogenic gene expression, and stimulates osteoblast activity via cAMP and Wnt/β-catenin signaling (Mrak et al., 2010). A decrease in VIP levels induces a concomitant increase (>50%) in osteoclast-covered surface in rat mandible and calvariae (Elefteriou, 2005). SP inhibition exacerbates bone loss via decreased MSC recruitment, as evidenced by increased osteoblast activity and decreased OPG/RANKL ratio in ovariectomized murine models of OP (Elefteriou, 2005). Adrenergic (NE) signaling directly stimulates osteoclast differentiation through upregulation of RANKL by binding β2AR, a β-adrenergic receptor highly expressed in osteoblasts (Brazill et al., 2019). Inhibiting DBH signaling lowers sympathetic tone, induces osteoblast proliferation, and increases mean BMD in murine bones (Elefteriou, 2005). Deletion of NPY and its major receptor, Y2, in selective knockout mice stimulates osteoblast activity and increases both cortical and trabecular bone formation (Baldock et al., 2002).

Given the above, that the hypothalamus enjoys a central role in regulating bone homeostasis comes as no surprise. Neural-osteo interplay appears to occur through two distinct channels: (1) well-defined hormonal signals generated in the hypothalamus and subsequently processed in the pituitary; (2) efferent neuronal discharges originating from the hypothalamus and processed through the brainstem (Driessler and Baldock, 2010). Chronic stimulation of sympathetic outflow is known to have detrimental effects on bone: indeed, sustained β2AR signaling on osteoblasts and osteocytes disrupts their capacity to maintain the endosteal HPSC niche. Various immune players have been implicated in regulating local neuropeptide secretion (Serre et al., 1999) and, under certain conditions, uptake (Pirzgalska et al., 2017), but further investigation is required to determine whether these mechanisms can be exploited for designing therapeutic interventions.

### Dysregulated Autophagic Homeostasis

Autophagic lysosome deficits occur early in AD onset and are hypothesized to be significant contributors to disease pathology (Zare-shahabadi et al., 2015). As early as 1967, abnormal aggregations of subcellular vesicles—subsequently identified as immature autophagic vacuoles—were reported to accumulate in dystrophic neurites in the AD brain (Suzuki and Terry, 1967). Aberrant lysosomal activity in AD resembles that induced by knocking out specific cathepsins or by administering lysosomal protease inhibitors. Prevailing theory suggests that failed protein and organelle catabolism by dystrophic autophagosomes induces a compensatory mechanism whereby autophagy is upregulated via ROS-dependent activation of type III PI3 kinase. Unfortunately, because downstream degradative pathways (chiefly lysosomal acidification) are already dysregulated, this only accelerates disease pathology. Promoting cathepsin activity via deletion of cystatin B (a cathepsin inhibitor) rescues autophagic-lysosomal pathology, reduces pathological Aβ accumulations, ubiquitinates proteins within autophagosomes, and reduces intraneural Aβ peptide (Yang et al., 2011). The pathological associations between dysregulated autophagic processes and neurodegeneration in AD are emphasized by the similar clinical features observed in certain lysosomal storage disorders: neurofibrillary tangles are seen in human Niemann Pick Type C disease and mucopolysaccharidosis type IIB (Ryazantsev et al., 2007). Further, evidence suggests that APOE-ϵ4, considered a risk factor toward the onset of sporadic AD, may work in concert with Aβ peptides to incite lysosomal membrane disruption, release of lysosomal enzymes, and subsequent neuronal degradation. While counterintuitive, global *inhibition* of autophagy, when deviant as in neurodegenerative disease, may be beneficial (Tung et al., 2012).

While the role of autophagy in the pathogenesis of age-related chronic inflammatory diseases like OP and OA requires elucidation, autophagic processes are intimately ingrained in the maintenance of bone and cartilage homeostasis. Increased autophagy is assumed critical in osteogenesis due to the requirement for rapid organelle recycling, preservation of nutrients, and the increased environmental susceptibility to hypoxia inherent to the osteoblast-to-osteocyte transition (Manolagas and Parfitt, 2010). In articular cartilage, primarily characterized by low cell turnover and limited vascularization, autophagy is essential for maintaining cellular integrity, function, and survival. Indeed, expression of ULK1, Beclin1, and LC3, an inducer, regulator, and executor of autophagy, respectively, was found to decrease with GAG loss in both age-related and surgically induced OA (Caramés et al., 2010). Importantly, autophagosome formation is heavily upregulated in the superficial and medial zones of OA cartilage in early disease stages and apoptotic factors dominate with disease progression, suggesting a shift toward an apoptotic phenotype that may be due, at least in part, to failed autophagy similar to that observed in AD (Almonte-Becerril et al., 2010).

Recent studies have purported autophagy inhibition as a novel treatment paradigm for inflammation-mediated osteoclastogenesis. Overall resorptive activity decreased in osteoclast monoculture following bafilomyocin (potent autophagy inhibitor) administration (Neutzsky-Wulff et al., 2010). These findings were later recapitulated in a murine model of bone loss induced by both ovariectomy and glucocorticoid treatment, where pharmacological (chloroquine) and genetic (*Atg7* deletion) suppression of autophagy in monocytes reduced osteoclastogenesis and subsequent bone resorption (Lin et al., 2016). Others, however, have reported the opposite: promoting autophagy in osteoblasts rescued viability following glucocorticoid treatment and reduced bone loss (Yao et al., 2016). Deletion of *FIP200* (involved in autophagosome formation) in osteoblasts induces osteopenia in rats (Yao et al., 2016). *Atg7* osteocyte knockout was shown to promote BMD loss in both male and female mice, not unlike that seen during natural aging (Onal et al., 2013). It appears that, overall, upregulation of autophagy in osteocytes and osteoblasts relieves oxidative stress, promotes cellular viability, and decreases bone resorption, while increased autophagy in osteoclasts exacerbates and accelerates bone and articular cartilage degradation in OP and OA. This precludes the use of systemic autophagy inhibitors for the treatment of these pathologies and underscores the need to develop vehicles for targeted stimulation or inhibition of autophagy in defined cell types. Moreover, the net effect of aging on autophagy on the microscopic scale requires further investigation: while age-related senescence contributes to a global reduction in autophagy (Caramés et al., 2010), the resulting accumulation of oxidative stress may induce autophagy predominantly in inflammatory mediators involved in disease pathology. A study delineating the propensity of different cell types toward increased autophagy following ROS stimulation at varied disease stages may provide some insight.

### Pathological MicroRNA Profiles

Micro ribonucleic acids (miRNAs) are sentinels of post-transcriptional regulation of gene expression: by binding the 3'-untranslated regions (UTRs) of their target genes, miRNAs prevent translation—either through direct translation suppression or mRNA cleavage (Llave et al., 2002). Due to the ubiquity of 3'-UTR motifs and the wide gamut of complementary microRNAs discovered in recent years, these short nucleotide strands are estimated to target and modulate expression of over 80% of all genes in humans (Herrera-Espejo et al., 2019). Dysregulation of miRNA profiles has thus garnered considerable interest as a prominent driving force of several systemic pathologies, including those discussed herein. Indeed, a host of miRNAs regulate genes involved in production of amyloid plaques (Jahangard et al., 2020) and hyperphosphorylated tau (Femminella et al., 2015; Moncini et al., 2017), as well as those encoding cytokines canonically associated with chronic neuroinflammation (Ravari et al., 2017; Liu et al., 2019)—most of which are downregulated in the AD brain (Reddy et al., 2017b). Multiple target genes implicated in the inception and maintenance of chronic peripheral inflammation (Zhu et al., 2012; Bogunia-Kubik et al., 2016) and concurrent cartilage degradation (Park S. J. et al., 2013) and osteopenia (Kelch et al., 2017) likewise continue to be evaluated. While dysregulated miRNA profiles in AD (Herrera-Espejo et al., 2019), RA (Reyes-Long et al., 2020), OA (Sondag and Haqqi, 2016), and OP (Ko et al., 2020) have been thoroughly reviewed elsewhere, Table 1 lists the miRNAs prominently referenced in recent literature, identifies whether they are up- or down-regulated in each condition, and provides a succinct overview of their respective targets and putative contributions to disease pathology. Inconsistencies in the expression of these miRNAs taken from different patient cohorts and procurement sites exemplify the complexity of miRNA biology: greater standardization and experimentation is required to uncover any direct correlation between those miRNAs differentially expressed in peripheral inflammatory bone and joint disorders and the onset and exacerbation of neurodegenerative disease.

**Table 1:** Dysregulated miRNA profiles implicated in the pathologies discussed herein.

**Disease**	**miRNA**	**Expression vs. Control**	**Relevant Target(s) || Putative contribution to pathology**	**Source(s)**
AD	9	Down	*FGFR1, SIRT1, REST* || Downregulation correlates directly with reduced cortical thickness and cognitive performance in AD patients	Kumar and Reddy, 2016; Maldonado-Lasuncion et al., 2019
	16-5p	Down	*APP* || Inhibition leads to accumulation of APP, subsequent dysregulation of insulin pathway and heighted expression of Raf and/or NF-κβ	Liu et al., 2012; Kirouac et al., 2017
	29	Down	*BACE1, BIM* || Expression inversely correlated with BACE1; Treatment with exogenous miR-29b has been shown to reduce expression of Aβ, and its pathological effects, *in vitro*	Jahangard et al., 2020
	34a-5p	Up	*p53* || Heightened miR-34 expression associated with tau hyperphosphorylation; Downregulation has been found to rescue some cognitive abilities in murine models	Zovoilis et al., 2011; Femminella et al., 2015
	106	Down	*APP, ABCA1* || Overexpression may inhibit amyloid-associated tau aggregation	Kim et al., 2012; Liu et al., 2016b
	107	Down	*CDK5* || Heavily downregulated in the hippocampus and temporal cortex of AD patients; CDK5 involved in tau hyperphosphorylation	Shukla et al., 2012; Moncini et al., 2017
	125-5p	Up	*DUSP6, PPP1CA, Bcl-W* || Upregulation associated with heightened neuroinflammation	Banzhaf-Strathmann et al., 2014; Reddy et al., 2017a
	132-3p	Down	*SIRT1, FOXO1, p250GAP* || Reduction in miR-132 appears preclude neuron loss; *in vitro* miR-132 protects neurons against both Aβ and glutamate; In early AD, expression is increased and correlated to higher MMSE scores; In late AD, expression is abrogated in both AD brain and neural exosomes	Wong et al., 2013; Hadar et al., 2018; Cha et al., 2019
	146a	Up	*TLR2* || Key regulator of AD-related immune response and implicated in multiple inflammatory pathologies including AD; Murine models demonstrate positive correlation between miR expression, senile plaque density, and cognitive impairment	Li et al., 2011; Ravari et al., 2017; Ansari et al., 2019
	155	Up	*c-Maf, IFNGR1, SHIP1* || Regulates microglial inflammatory response; Heavily upregulated in 3xTg murine models of AD; elevated levels coincide with c-Jun expression, microglial and astrocyte activation, and upregulated secretion of inflammatory mediators; Has also been implicated in activation of a wide gamut of T lymphocytes	Song and Lee, 2015; Liu et al., 2019; Zhao et al., 2019
	181a/c/d	Down	*SPTLC1, c-Fos, SIRT1* || Regulates cell proliferation, apoptosis, autophagy, mitochondrial function, and immune response; Loss increases serine palmitoyltransferase	Ouyang et al., 2012; Rodriguez-Ortiz et al., 2014; Indrieri et al., 2020
	212-3p	Down	*SIRT1* || Correlations similar to those observed in 132-3p	Hadar et al., 2018
RA	16-5p	Up	*A2AaR* || Results in upregulation and activation of NF-κβ pathways; upregulation in Th17 cells; treatment with anti-TNF agents or DMARDs led to significantly increased expression	Reyes-Long et al., 2020
	23b-3p	Up/Down	*NOX4, TAB2, TAB3, IKK-*α || Immunosuppressive via regulation of NOX4, which in turn inhibits expression of proinflammatory cytokines COX2, TNF-a, and IL-1β; Shown to be protective of GABAergic and motor neurons; Regulates NF-κβ via TAB2, TAB3, and IKK-a (genes through which TNF-α, IL-17, IL-1β activate the NF-κβ pathway); IL-17 creates dysregulated feedback loop between 23b-3p and NF-κβ, leading to increased expression of both	Zhu et al., 2012
	124-3p	Up/Down	*I*κβ*, MCP-1, SIRT1* || Pathological downregulation leads to repression of inhibitors of κβ (iκβ), ultimately increasing NF-kB expression	Chiu et al., 2020
	146-5p	Down	*TRAF6, JNK/CCL2, NF-*κβ || Serum expression significantly reduced in RA patients compared to controls, but found significantly increased in synovial tissue and synovial fluid-derived monocytes; expression induced by TNF-α and IL-1β	Bogunia-Kubik et al., 2016; Reyes-Long et al., 2020
	155-5p	Up	*SOCS1* || Overexpressed in synovial joints of RA patients, leading to suppression of SOCS1 and triggering expression of TNF-α and IL-6	Bogunia-Kubik et al., 2016
	223-3p	Up	*E2F1* || Predominantly expressed in Th cells; Overexpression in RA decreases E2F1 levels, leading to dysregulation of T-lymphocyte phenotype and subsequent autoimmunity	Pawlik et al., 2003; Fulci et al., 2010
OA	9	Down	NF-κβ || Overexpression has been implicated in reduction of NF-κβ pathway signaling factors including NF-κβ, TNF-α, IL-1β	Bazzoni et al., 2009; Liu et al., 2016a
	34a	Up	*SIRT1* || Upregulation results in concomitant decrease in SIRT1 expression; injection of a lentiviral vector encoding anti-miR-34a effectively abrogated OA progression in rat models	Yan et al., 2016
	130	Down	*TNF* || Downregulated in OA patients, with concomitant upregulation in TNF	Li et al., 2015; Panagopoulos and Lambrou, 2018
	146a	Up	*CAMK2D, PPP3R2* || Upregulation exacerbates proinflammatory cytokine secretion; miR-146a overexpression murine model demonstrated significantly higher TNF-α, IL-1β expression; TNF-α, IL-1β, and IL-17 administration appear to elevate miR levels in a positive feedback loop manner	Zhang X. et al., 2017
	149	Down	*MyD88, STAT3* || Effective modulator of a wide variety of pro-inflammatory factors; inhibits hepatic inflammatory response via STAT3 pathway; Overexpression in macrophages linked to inhibition of NF-Kb, TNF-a, and IL-6	Xu et al., 2014; Zhang Q. et al., 2017; Tahamtan et al., 2018
	199	Down	*SMAD1, MAPK* || Involved in promotion of chondrogenesis; Promotes osteoblastic differentiation of hMSCs via down- and up-regulation of SOX9 and aggrecan, respectively; Downregulation in OA results in increased expression of COX-2	Laine et al., 2012; Zhang et al., 2012
	558	Down	*COX2* || Directly suppresses COX-2 mRNA activity and IL-1β induced catabolic events in chondrocytes to promote homeostasis	Park S. J. et al., 2013
OP	9-5p	Up	WNT3A || Found to be highly expressed in OP patients relative to negative controls; Promotes adipogenesis and inhibits osteogenesis	Zhang H. G. et al., 2019
	21	Up/Down	*PDCD4* || Upregulated in senile osteoporosis, which leads to c-Fos expression and subsequent osteoclastogenesis; Role in osteoblast differentiation is more controversial	Sugatani et al., 2011; Cheng et al., 2019
	23-3p	Up	*RUNX2-SATB2* || Inhibition of SATB2 expression may incite osteoclastogenesis	Yavropoulou et al., 2020
	29b	Down	*HDAC4, RUNX2* || Decrease in miR-29b causes concomitant increase in HDAC4 and subsequent reduction in osteoblastic differentiation	Li Z. et al., 2009; Bellavia et al., 2019
	100	Up	*BMPR2, SMAD1* || Inhibits osteogenic differentiation of MSCs; *Ex vivo* study of osteoclasts taken from OP patients revealed an inverse correlation between miR-100 expression and BMD at the femoral neck	Fu et al., 2016; Bellavia et al., 2019
	124	Up	*Dlx2, Dlx3, Dlx5* || MiR-124 overexpression has been shown to drive MSC adipogenesis; Significantly elevated in patients with low bone mass	Qadir et al., 2015; Yavropoulou et al., 2017
	125	Up	*CBF*β || Inhibition of osteogenesis via RUNX2 suppression; MiR-125 level found to be inversely correlated with patient femoral head BMD; Circulating miR-125 found to be significantly upregulated in osteoporotic patients	Huang et al., 2014; Liu et al., 2015; Panach et al., 2015
	133	Up	*RUNX2, CXCL11, CXCR3, SLC39A1, TCF-7* || Suppression of osteoblastic differentiation via RUNX2 inhibition	Li et al., 2008; Wang Y. et al., 2012; Liao et al., 2013
	187	Down	*IL6, TNF* || Elevated expression may lead to increased pro-inflammatory cytokine expression and inhibited osteogenesis	Garmilla-Ezquerra et al., 2015
	2861	Down	*HDAC5* || Significantly downregulated in osteoporosis patients, leading to HDAC5-mediated inhibition of RUNX2 and BMD loss	Li H. et al., 2009

## Mesenchymal Stem Cell (MSC) Therapy

**Figure 4:**
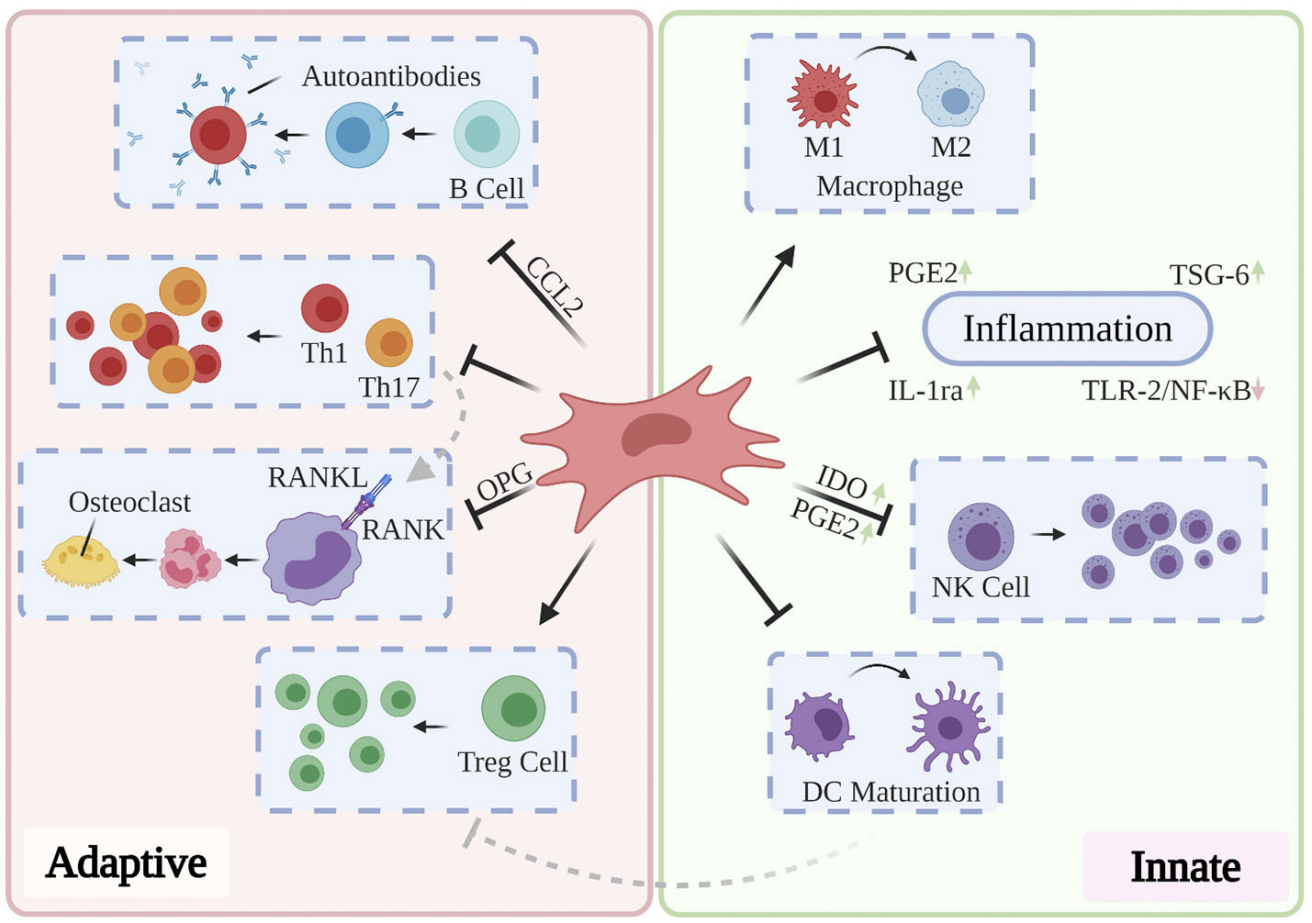
Partly hypothetical model for the key immunosuppressive mechanisms of MSCs, which modulate the activation, proliferation, and function of prominent mediators of both the innate and adaptive immune systems. In addition to quelling production of local inflammatory cytokines via secretion of PGE2, IL-1ra, and TSG-6, primed MSCs have been shown to reverse the pro-inflammatory polarization of macrophages, inhibit proliferation of NK cells via IDO and PGE2, and prevent the maturation of DCs. Local and systemic administration of MSCs has also been shown to restore normal Th1/Th17:Treg ratios, prevent the production of pathogenic autoantibodies via CCL2, and inhibit osteoclastogenesis through OPG production. These and other findings contribute to the hypothesis that MSCs are uniquely suited to treat a variety of chronic inflammatory diseases.

Cumulatively, the results of the above studies suggest that effective treatment of RA, OA, and OP may delay and ameliorate AD-related neurodegeneration and that they may do so through targeting mechanisms associated with aging. The limited longitudinal clinical benefits conferred by the currently administered pharmacological and antibody-based therapies highlights the need to investigate novel paradigms for the treatment of these disorders. Moreover, while conservative, systemic anti-inflammatory treatments may suffice for short-term improvement in patient-reported pain and range of motion, their role in ameliorating the underlying structural abnormalities in bone and cartilage remain limited (Jevotovsky et al., 2018). The introduction and usage of stem cells represents an important advance in regenerative cellular therapy: a number of works report preclinical benefits through differentiation of induced pluripotent stem cells (iPSCs) and embryonic stem cells (ESCs) into targeted cell types and these findings have been thoroughly reviewed elsewhere (Burke et al., 2016; Duncan and Valenzuela, 2017); however, their tendency to incite teratoma growth (Zakrzewski et al., 2019) as well as their immunogenicity (Deuse et al., 2019) has precluded their mainstream usage. MSCs possess excellent therapeutic potential for a broad range of chronic inflammatory and neurodegenerative conditions, owing to their accessibility relative to embryonic and induced pluripotent stem cells, their relatively predictable behavior, and their inherent ability to differentiate into osteoblasts, chondrocytes, and adipocytes. Typically isolated from bone marrow, adipose tissue, and more recently, the umbilical cord (Xu et al., 2018), MSCs mediate wound-healing by exerting pro-angiogenic, anti-fibrotic, and anti-inflammatory activity through direct cell-cell interactions and via the secretion of potent trophic factors (Shi et al., 2018). In addition to retaining oxidative stress resistance in inflammatory environments (Cui et al., 2017), MSCs modulate the activation, proliferation, and function of key mediators of both the innate and adaptive immune systems (see Figure 4). As such, they are uniquely suited to serve as the foundation for multiple therapies designed to ameliorate AD-related neurodegeneration, chronic systemic inflammation, and arthritis-associated bone and cartilage degradation. Over past decades, a plurality of studies have found that direct intracerebral (AD) and intraarticular (RA, OP, OA) injection of MSCs confers multiple benefits evidenced by three key features: (1) inflammation remission, (2) stimulation of neotissue formation, and (3) measurable improvements in behavioral outcomes. While these investigations are thoroughly reviewed elsewhere (Duncan and Valenzuela, 2017; Kim and Shon, 2020), we here discuss current developments toward maximizing the clinical usability and therapeutic potential of MSCs as they apply to AD, inflammatory arthritis, and OP.

### Systemic MSC Injection

Systemic or intravenous (IV), MSC administration confers a key clinical benefit: minimization of the proximal tissue damage inherent to local MSC injection boluses. This coupled with accumulating evidence that intravenously injected MSCs home to sites of interest (Sui et al., 2016) and retain therapeutic efficacy comparable to direct injection routes (Cui et al., 2017; Harach et al., 2017) renders systemic administration an appealing and viable treatment paradigm. In AD, intravenously injected MSCs have been shown to traverse the BBB and, importantly, display no evidence of eliciting a tumorigenic or immune response (Duncan and Valenzuela, 2017). While the mechanisms by which MSCs exert their therapeutic potential in the AD brain have yet to be clarified, multiple studies have shown that MSCs can differentiate into a plurality of neural cell types and enhance neurogenesis through the secretion of neurotrophic factors (Park D. et al., 2013; Garcia et al., 2014; Kim et al., 2015). Recent animal studies reflect this paradigm: intravenous transplantation of 2 × 10^6^ human umbilical cord (hUC)-MSCs into 12 month-old Tg2576 mice improved cognitive performance as assessed by the Morris water maze 4 weeks after transplantation, attenuated oxidative stress, promoted neuronal proliferation, supported neurogenesis in the hippocampus, and increased expression of neurotrophic factors Sirt1, brain-derived neurotrophic factor (BDNF), and α-synuclein (SYN) (Cui et al., 2017). APP/PS1 mice given an equivalent treatment of bone marrow (BM)-MSCs demonstrated a significantly reduced escape latency in the Morris water maze, decreased concentrations of pathological Aβ_1−42_ and beta-secretase 1 (BACE1), and attenuated expression of inflammatory cytokines IL-1, IL-2, TNF-α, and IFN-γ in whole blood samples (Wei et al., 2018). Collectively, IV MSC treatment appears to ameliorate cognitive dysfunction by promoting neurogenesis and synaptic plasticity, increasing secretion of neurotrophic factors, decreasing hippocampal oxidative stress, and modulating expression of Aβ-related genes.

Similarly encouraging results have emerged for its application in inflammatory arthritis. In a Phase Ia clinical trial including predominantly post-menopausal women, IV injection of 1 × 10^8^ hUC-MSCs reduced whole blood levels of IL-1β, IL-6, IL-8, and TNF-α 24 h post-injection (Park et al., 2018). In another controlled trial including 53 patients, nearly 50% of those receiving a single injection of autologous adipose-derived (A)-MSCs achieved ACR20—a clinical benchmark for treatment efficacy—within the first month; however, benefits were found to diminish after 3 months, suggesting longitudinal efficacy would require repeated treatments (Álvaro-Gracia et al., 2017). In a macaque model of OA receiving 2 weekly injections of 1 × 10^7^ allogenic MSCs, immunohistochemical staining revealed that the peripherally administered cells localized in and around the injured synovium (Fernandez-Pernas et al., 2017). Surprisingly, proliferation and activation of endogenous MSCs was heavily upregulated 2 weeks post-infusion, suggesting injected MSCs exert their therapeutic affects partly by recruiting and activating endogenous senescent MSCs. Finally, systemic infusion of 1 × 10^6^ allogenic BM-MSCs via the caudal vein maintained trabecular bone mass in glucocorticoid-challenged murine models of OP and promoted osteoblast and osteoprogenitor survival (Sui et al., 2016). As with previous studies, donor MSCs were found to specifically home and engraft to recipient bone marrow 4 weeks post-infusion.

Despite these encouraging findings, systemic MSC injection is not free of limitations. While IV administration is less invasive than established local injection paradigms and allows for the therapeutic cells to disseminate throughout the body, significant pulmonary MSC entrapment has been observed in a number of animal models (Fischer et al., 2009; Ankrum and Karp, 2010; Zheng et al., 2016). Before they attain systemic circulation, MSCs pass through and agglutinate in the lungs, largely due to interactions between the abundance of pulmonary fibronectin and vitronectin, and select adhesion integrins on the MSC surface (Wang S. et al., 2015). While promising advancements have been made in mitigating this phenomenon—be it via antibody-mediated integrin blockade (Wang S. et al., 2015) or strategic culturing practices during the *in vitro* expansion of MSCs from select sources (Nystedt et al., 2013)—pulmonary MSC entrapment represents a significant clinical obstacle which merits further investigation.

### MSC Conditioned Medium

While MSCs exert potent immunosuppressive functions following exposure to an inflammatory microenvironment (Noronha Nc et al., 2019), recent studies suggest that longitudinal interactions with pro-inflammatory cytokines and their mediators may gradually reduce their clinical efficacy (Shi et al., 2018). Researchers have identified several attributes inherent to MSCs that limit their therapeutic efficiency in injections, including low survival rates in pathological microenvironments and the concomitant requirement for substantial overexpansion prior to injection, and considerable variability in donor properties, *in vitro* culture conditions, and clinical performance assessment procedures (Noronha Nc et al., 2019). These and other deficiencies have prompted investigation into the purely paracrine modality of MSC-mediated immunosuppression through utilization of MSC-conditioned medium (MSC-CM). Conditioned medium extracted from primed MSCs presents several hypothetical advantages: (1) CM can be manufactured in tightly controlled *in vitro* culture conditions optimized for mass production; (2) It can be freeze-dried, packaged, and subsequently transported far easier than live MSC populations; (3) A single CM batch preparation can be used for multiple therapeutic injections; and (4) CM vastly decreases the probability of host rejection and the aberrant immune response inherent to allogenic stem cell transplantation (Chen et al., 2018).

The results of numerous animal studies support the clinical efficacy of MSC-CM. Sustained microglial activation has been implicated in the pathogenesis and exacerbation of AD (Colonna and Holtzmann, 2017; Perea et al., 2018). Both murine carcinoma (BV2) and primary human microglia showed a ~50% reduction in the secretion of pro-inflammatory cytokines TNF-α and IL-6 and increased IL-10 production following LPS activation when cultured in MSC-CM for 24 and 6 h, respectively (Ooi et al., 2015). A separate study found that MSC-CM protected BV2 microglia from Aβ_35−45_ challenge by reducing BV2 proliferation and apoptosis, promoting Aβ phagocytosis, correcting aberrant autophagic profiles, and upregulating expression of Aβ-degrading enzymes (Xu et al., 2018). Intraarticular injection of concentrated MSC-CM into antigen-induced arthritis (AIA) murine models reduced TNF-α sera concentration, attenuated aggrecan breakdown, increased production of IL-4 and FOXP3, and restored Treg:Th17 balance (Kay et al., 2017). Incubation of LPS-activated chondrocytes in concentrated MSC-CM decreased transcription of proinflammatory genes at both 24 and 72 h post-treatment, increased expression of ECM markers AGG and COL1, and increased global chondrocyte viability relative to untreated controls (Chen et al., 2018). Intriguingly, multiple reports have proposed that MSC-CM can induce a similar or stronger osteogenic effect than transplanted cells (Osugi et al., 2012; Chen et al., 2018). *In vivo* imaging and immunohistopathological staining of transgenic OP rats revealed that MSC-CM treatment groups displayed a larger area of newly regenerated bone and greater recruitment of native MSCs to the defect area compared to MSC-injected groups (Osugi et al., 2012). This finding adds further credence to the hypothesis that MSCs exert their regenerative effects partly through the mobilization of endogenous stem cells.

In the short term, MSC-CM exerts powerful neuroprotective, chondroprotective, and anti-inflammatory effects; however, the relatively short experimental timepoints of the above works (~3–7 days) highlight the need for elucidating the longitudinal effects of MSC-CM treatment, dose requirements, and treatment frequency to produce optimal therapeutic outcomes. Moreover, tightly regulated manufacturing standards (e.g., basal media formulations, MSC incubation period, MSC seeding density, MSC age, donor, etc.) must be enforced to rigorously test clinical efficacy. Isolation and utilization of MSC-derived exosomes further diminishes potential immunogenicity concerns associated with MSCs and their derivatives. Indeed, MSC-secreted exosomes have recently been found to orchestrate—to a significant degree—MSCs' therapeutic mechanisms of action. This exciting field of MSC therapy has been thoroughly reviewed elsewhere (Mendt et al., 2019; Yin et al., 2019; Forsberg et al., 2020).

### Biomaterial-Based Approaches

The field of tissue engineering is dominated by two primary strategies for creating regenerative tissue constructs: scaffolds and spheroids. While spheroid architectures intrinsically promote cell-cell interactions and cellular fusion into cohesive constructs that endogenously produce ECM, they often demonstrate inadequate mechanical properties, especially when used to regenerate load-bearing tissues. Alternatively, scaffolds are suitable for applications requiring compressive and torsional strength, native cellular infiltration, and neotissue deposition. In addition, scaffolds are remarkably versatile, enabling a broad range of mechanical and degradative properties, and can be tailored to release therapeutic molecules either via controlled release or surface immobilization (McMasters et al., 2017; Ovsianikov et al., 2018). Finally, combinatorial approaches utilizing multiple substrates afford advanced characteristics like shape-memory and endogenous induction of targeted cell phenotypes. Engineered scaffolds thus represent an appealing paradigm for maximizing the therapeutic efficiency of MSCs, either through directed differentiation or stimulation of immunosuppressive phenotypes.

#### OP

Electrospun gelatin scaffolds, which demonstrate structural properties similar to native collagen, have been shown to promote MSC proliferation, survival, and osteogenic differentiation in the absence of exogenous growth factors (Chang et al., 2012). Following 21 days of osteogenic induction, BM-MSCs seeded in pure gelatin scaffolds demonstrated significantly increased mineralization relative to 2D controls (Moll et al., 2017). Poly(ϵ-caprolactone) (PCL) is used commonly in general scaffold design owing to its biocompatibility, biodegradability, low immune reactivity, optimal biomechanical properties and the ability to form complex 3D shapes; however, its usage in osteogenic induction is limited as it lacks the surface reactivity necessary for cell attachment. Application of composites like hydroxyapatite, and more recently, powdered oyster shells (OS), overcome these detriments by conferring hydrophilicity and topographical variance. Seeding MSCs on OS-coated PCL scaffolds enhanced MSC proliferation, significantly promoted osteogenic differentiation, increased long-term MSC viability, and demonstrated higher levels of alkaline phosphatase (ALP) activity and calcium deposition than bare PCL scaffolds (Didekhani et al., 2020).

Numerous studies have reported that applying HA coating to polymer meshes supports osteoblast function and osteogenic differentiation (Sato et al., 2006; Nguyen et al., 2013; Venugopal et al., 2013). The application of HA nanoparticles to electrospun PCL scaffolds dramatically increased hydrophilicity in the absence of plasma treatment, increased ALP activity by 20% compared to PCL/collagen controls, markedly increased mineralization, and incited noticeable changes in cellular morphology associated with osteogenesis (Venugopal et al., 2013). Indeed, HA and other major bone constituents including α- and β-tricalcium phosphate (TCP) are the most widely investigated ceramic scaffold supplements for osteogenic stimulation of MSCs; however, both lack vital elements related to bone metabolism and TCP generates alkaline degradation products that lead to proximal cytotoxicity and reduction in scaffold mechanical properties. Zinc-containing hardystonite (HS) has been shown in PCL scaffolds to surpass HA coating in mechanical strength, MSC proliferation, scaffold infiltration, ALP activity, and mineralization (Jaiswal et al., 2013). These effects were later recapitulated in electrospun PLLA scaffolds, which demonstrated increased osteonectin and OCN expression (Tavangar et al., 2018). Other studies have indicated that HA and TCP supplementation with various phytocompounds enhances their osteogenic effects: incorporation of 20 μM diosmin augmented ALP activity and calcium deposition, and increased expression of RUNX2, ALP, COL1, OCN, and osterix following 14 days of culture (Chandran et al., 2019). After 11 days, 10^−8^–10^−6^ M icariin increased ALP expression and bone mineralization (Fan et al., 2011). 5–10 μM chrysin enhanced ALP activity, produced a marked increase in mineralization and calcium deposition, and sustained upregulation of RUNX2 expression (Menon et al., 2018).

#### OA

The mechanical stimuli produced in tissue microenvironments is known to direct differentiation of stem cells to terminal, specific fates. Hydrogel systems have demonstrated an excellent capacity to enhance chondrogenesis of MSCs by approximating the fibrous nanostructure of articular cartilage, but they are limited in their ability to simultaneously recapitulate the most crucial physiological properties of cartilage: compressive and viscoelastic moduli, porosity, and complex *in vivo* geometry. To illustrate, while the compressive modulus of human articular cartilage ranges from 240 to 1,000 kPa, that of typical hydrogel systems is lower by at least an order of magnitude (Beck et al., 2016). Bioengineers and material scientists are thus often presented with a “tightrope walk,” where they must balance the high viscoelastic moduli needed to intrinsically promote chondrogenesis with requisite scaffold porosities enabling effective seeding, native cell perfusion, and nutrient diffusion. To that end, Aliabouzar et al. tested multiple pore geometries and found that small (700 × 690 μm) square pores stimulated significantly higher MSC proliferation than hexagonal pores (Aliabouzar et al., 2018). This finding coincided with those of other investigations which indicated that MSCs preferentially adhere to and proliferate more rapidly on larger curvatures (Knychala et al., 2013; Zhou et al., 2016).

In a recent study, various popular polymer chemistries were evaluated for their inherent potential to direct chondrogenic differentiation in seeded MSCs. Of the six formulations tested, poly-L-lysin-coated polydioxanone (PDO) and poly-L-ornithine (PLO) scaffolds best supported chondrogenic fate commitment, resulting in increased sulfated GAG concentration and chondrogenic matrix deposition. Notably, these effects persisted in the absence of supplemented chondrogenic growth factors (San-Marina et al., 2017). Scaffolds are frequently implanted to sequester therapeutic cells to injury sites. Poly-lactic-co-glycolic acid (PLGA) nano fibers (NFs) promote MSC proliferation and differentiation into osteoblasts under osteogenic culture conditions. Immunohistology revealed that MSCs seeded in columnar-shaped PLGA NFs enjoyed greater chondrogenic potential compared to 2D controls in equivalent media, as shown by heavily upregulated SOX9 and COL10A1 mRNA expression (Sonomoto et al., 2016).

#### AD

Intracranially transplanted cells typically generate sparse amounts of non-neuronal cells or unexpectedly die (Menon et al., 2018). Additionally, limitations in real-time imaging and human error often produce spatial disparities between injury and therapeutic injection sites. Although there currently exists no published literature pertaining to scaffold-based tissue regeneration in AD, various pre-clinical studies in models of related neurodegenerative disorders have produced encouraging results. Collagen scaffold implantation of MSCs in a rat model of TBI improved cell survival and neurite outgrowth *in vivo*, limited distribution of MSCs to the transplanted region, improved brain metabolism, and resulted in improved neurite functional recovery compared to direct MSC injection (Menon et al., 2018). Following surgical resection-simulated chronic TBI in rats, fibrous collagen scaffolds obtained from bovine aponeuroses and seeded with hUC-MSCs improved locomotion, promoted neural regeneration and remyelination, induced proximal neurogenesis, and blocked astrocyte proliferation outside the lesion area (Wang et al., 2018). Self-assembling nanofiber scaffolds (SANs), characterized by a repetitive peptide sequence which mimics native ECM properties, have garnered recent attention in AD therapeutics owing to their inherent capacity to augment cellular adhesion, enhance axon growth impacts, and stimulate synapse development (Liedmann et al., 2012). Perhaps most importantly, SANs have been shown to innately interfere with APP processing by inhibiting BACE1 signaling, reducing the expression of Aβ_1−40_ and Aβ_1−42_ in APP/PS1 transgenic mice.

## Alternative Treatment Paradigms

As of this review, MSCs are a predominant non-pharmacological therapy applied toward peripheral chronic inflammatory conditions including RA, OA, and OP. The plurality of investigations reviewed herein support their usage, owing to their tissue regenerative properties and their intrinsic propensity to drive inflammation remission; unfortunately, current MSC procurement and administration methods limit their therapeutic efficacy and pose multiple clinical challenges. Firstly, the accepted clinical procedure for the harvesting and intra-articular injection of autologous MSCs is invasive—often entailing a prolonged recovery period (Harrell et al., 2019). Moreover, MSC injections are only approved for those with severe disease. When the procedure is ultimately approved, IV-injected MSCs have been shown to home to therapeutic areas of interest (Sui et al., 2016); however, undesired cell scattering is inevitable and degrades therapeutic efficiency (Zheng et al., 2016). Finally, few of the clinical studies enumerated above involve sufficiently long investigatory timepoints and thus fail to assess the longitudinal (>6 months) effects of MSC administration.

While drawing attention to the prevailing need to investigate novel methods for maximizing the clinical utility of MSCs is a focus of this review, a plurality of other therapeutic paradigms are under continual development and demonstrate tremendous potential for treating and/or modeling the complex, multifactorial conditions described herein. Following their discovery in the seminal works of Takahashi et al. (2007), iPSCs have contributed to astounding advancements in personalized medicine, disease modeling, and cellular therapy; indeed, iPSCs have been used as model system and treatment paradigm for AD (Devineni et al., 2016; Tcw, 2019; Penney et al., 2020), RA (Cassotta et al., 2020), OA (Dubey et al., 2018; Nakayama et al., 2020); and OP (Paspaliaris and Kolios, 2019; Rana et al., 2019). iPSCs retain several theoretical advantages over MSCs when applied to cellular therapies: in addition to being capable of unrestrained growth, they demonstrate relatively minimal immunogenicity and can be differentiated into a wide gamut of specific cell niches. When coupled with recent advances in genome editing, iPSCs enable interrogation of the consequences of various genetic and environmental perturbations in tightly controlled settings. While their embryo-derived counterparts, ESCs, represent the ideal source for cellular differentiation studies, they are generally considered unsuitable for clinical treatments due to their low differentiation efficiency, observed overgrowth within tissue grafts, tumorigenicity, and the host of ethical and safety concerns associated with their procurement and usage (Rana et al., 2019). Interestingly, iPSC-derived MSCs have gained abundant interest in recent years, as they integrate the benefits of both iPSCs and MSCs: autologous somatic cells can be harvested via relatively non-invasive procedures, and differentiated MSCs ostensibly confer immunosuppression and tissue regeneration with minimal risk of eliciting a host immune response (Khan et al., 2019).

Systems for targeted genomic modification have likewise gained considerable traction—particularly after the discovery of the Clustered Regularly Interspaced Short Palindromic Repeats (CRISPR)-Cas9 paradigm (Jinek et al., 2012). The Cas9 endonuclease, when paired with a single guide (sg)RNA, can efficiently cleave specific sites of double-stranded (ds)DNA, ultimately rendering specific gene segments inert (Lino et al., 2018). These gene knock-out models have been employed extensively toward refining searches for genetic disease susceptibility loci. In preclinical models of AD, most CRISPR-Cas9 mediated therapies are directed toward inhibiting the neurotoxic form of Aβ proteins and deactivation of γ-secretase activating protein (Karimian et al., 2020), and have demonstrated encouraging results toward their therapeutic application in both FAD and LOAD. The CRISPR-Cas9 system has also been utilized to generate numerous knockout models of inflammatory arthritis and OP. Following the identification of putatively pathological gene segments via genome-wide association studies, these genes of interest are either deactivated, modified, or spliced into model systems (often incorporating iPSCs) to faithfully recapitulate the diseased phenotype in controlled *in vitro* and *in vivo* settings—these findings have been thoroughly reviewed elsewhere (Adkar et al., 2017; Ding and Orozco, 2019; Wu et al., 2019; Yuan et al., 2019b). The simplicity, low cost, and high cleavage efficiency of CRISPR-Cas9 compared to earlier Transcription Activator-Like Effector Nuclease and Zinc Finger Nuclease based systems is appealing; however, its limitations merit serious consideration and currently preclude its use in mainstream clinical settings. Firstly, Cas9 is known to generate off-target modifications: a few mismatches distal to the protospacer adjacent motif do not prevent activation of the CRISPR-Cas9 system. Screening alternative Cas orthologs with enhanced specificity and target range has been attempted to mitigate this artifact (Wu et al., 2019). Founder mosaicism is another boundary: in knockout and transgenic models of disease, CRISPR-Cas9 components are injected into the fertilized zygote and continuously target and cleave genes during embryonic development, often causing mosaicism in the introduced mutations. Certain strategies are being investigated to combat mosaicism, including quickening the editing process (introducing Cas9 at very early zygote stages), shortening the longevity of Cas9, and CRISPR-mediated germline modification (Mehravar et al., 2019); these investigations are ongoing.

The conditions discussed in this review are complex and multifactorial, resulting from a wide array of genetic, epigenetic, and environmental factors; however, given that specific gene segments have been shown to drastically increase the risk and severity of these conditions, gene therapy has recently emerged as another exciting avenue of research. Gene therapy entails the delivery of therapeutic genes via specialized carriers, or vectors, that are typically viral, polymeric, or lipid-based in design (Deviatkin et al., 2020). Viral vectors have historically enabled remarkably stable and longitudinal transgene expression, but also demonstrate a host of safety concerns, including the risk of insertional mutagenesis inherent to retroviruses and activation of innate and adaptive immune mechanisms. Polymeric and lipid-based vector systems effectively eliminate these safety concerns, but are generally overlooked due to their comparatively low induction of transgene expression (Young et al., 2020). As with CRISPR-mediated treatments, the vast majority of gene therapy strategies for AD involve inhibition of the pathological variants of Aβ peptides, either through functional gene knockouts, Aβ immunization, or viral-mediated overexpression of genes encoding for enzymes that efficiently degrade Aβ (Choong et al., 2016). Adeno-associated viruses (AAVs) are the most popular vehicles for genetic therapies, owing to their proven efficacy and safety in a large number of animal models (Naso et al., 2017). Indeed, AAV-mediated enforcement of osteogenic and chondrogenic gene overexpression in transplanted somatic cells and infusion of exogenous recombinant growth and survival factors appears to be the focus of current clinical investigations in arthritis (Deviatkin et al., 2020; Young et al., 2020) as well as OP (Ball et al., 2018). Unfortunately, the results of multiple preclinical and clinical trials have made it yet unclear whether viral-mediated delivery of growth/survival factors is beneficial in these conditions (Ball et al., 2018; Honig, 2018; Deviatkin et al., 2020). Nonetheless, these drug and gene delivery vehicles—in conjunction with CRISPR-Cas9 and iPSCs—enable the development of combinatorial therapeutic strategies that bring us ever closer to truly personalized medicine.

## Conclusion

Accumulating evidence from preclinical, clinical, systematic, and meta-analysis studies reports that peripheral chronic inflammatory conditions including rheumatoid arthritis, osteoarthritis, and osteoporosis may contribute to AD pathogenesis and exacerbate inflammatory neurodegeneration with disease progression. While the mechanisms underlying these disease pathologies remain elusive, chronic inflammation is clearly implicated as a predominant driving force of bone, cartilage, and neuron degeneration, and numerous immunosuppressive therapeutic agents have produced positive clinical outcomes in AD—especially when administered prior to the onset of cognitive impairment. The chronic systemic inflammatory conditions discussed herein are prevalent—crucially, even among the young—and therefore merit serious consideration as significant factors contributing to AD pathogenesis. Moreover, the limited clinical success of treatments geared toward canonical AD targets in the CNS illustrates the need to consider more holistic approaches toward generating interventional therapies. Given the above data, we submit that effective treatment of these prominent peripheral immune disorders in early- to mid-life may significantly decrease the risk of and ameliorate inflammation-mediated cognitive decline in AD. MSCs are uniquely suited to serve as the foundation for multiple therapies designed to address these aberrant aging-related inflammatory conditions due to their robust immunosuppressive properties, their unique ability to activate and recruit senescent cells, and their excellent accessibility relative to their embryonic and pluripotent stem cell counterparts. Nonetheless, current administration methods limit their clinical and longitudinal efficacy, and increase the risk of donor site morbidity and host rejection of transplanted allogenic cells. Future studies, therefore, should investigate methods of maximizing the therapeutic efficiency of MSCs and their conditioned medium via isolation and concentration of select paracrine factors and combinatorial biomaterials to improve MSC localization to diseased regions of interest. Toward this end, decoupling the direct cell-cell and paracrine mechanisms by which MSCs exert immunomodulation is crucial. Emerging technologies and treatment paradigms such as CRISPR-Cas9 and vector-mediated gene therapy should be utilized in conjunction with MSCs to generate combinatorial, personalized therapies. Indeed, a holistic consideration of the contributions of peripheral immune processes to changes in the CNS may incite a paradigm shift in our understanding of AD pathogenesis and treatment.

## Author Contributions

RC performed all data acquisition, interpretation, and manuscript drafting. MH assisted with data interpretation and performed manuscript revision. All authors contributed to the article and approved the submitted version.

## Conflict of Interest

The authors declare that the research was conducted in the absence of any commercial or financial relationships that could be construed as a potential conflict of interest.

## Glossary

ABCA1, ATP-binding cassette transporter sub-family A; ACE-R, Addenbrooke's Cognitive Examination Revised; ACPA, Antibodies to citrullinated protein antigens; AD, Alzheimer's Disease; ADAMTS, Disintegrin and metalloproteinase with thrombospondin motifs; AGG, Aggrecan; AIA, Antigen-induced arthritis; ALP, Alkaline phosphatase; A-MSC, Adipose-derived mesenchymal stem cell; APOE, Apolipoprotein E; APP, Apolipoprotein; BACE1, Beta-secretase; BBB, Blood-brain barrier; BDNF, Brain-derived neurotrophic factor; BIM, Bcl-2-like protein 11; BMD, Bone mineral density; BM-MSC, Bone marrow-derived mesenchymal stem cell; CCL, C-C motif chemokine ligand; CD##, Cluster of differentiation ##; CDK5, Cyclin dependent kinase 5; CIA, Collagen-induced arthritis; CNS, Central nervous system; Col1, Collagen-1; CSF, Cerebrospinal fluid; CXCL, C-X-C motif chemokine; CXCR; C-X-C motif receptor; DAM, Disease-associated microglia; DAMP, Damage-associated molecular pattern; DBH, Dopamine beta-hydroxylase; DMARD, Disease-modifying anti-rheumatic drugs; ECM, Extracellular matrix; FGFR1, Fibroblast growth factor receptor 1; GABA, Gamma aminobutyric acid; GCRP, Calcitonin gene-related peptide; HA, Hydroxyapatite; HMGB1, High-mobility group box 1; HPSC, Hematopoietic progenitor cell; HS, Hardystonite; hUC-MSC, Human umbilical cord-derived mesenchymal stem cell; IFN-γ, Interferon gamma; IL, Interleukin; IV, Intravenous; IκB, Inhibitor of nuclear factor κB; LBD, Lewy body dementia; LPL, Lipoprotein lipase; LOAD, Late-onset Alzheimer's Disease; LPS, Lipopolysaccharide; MAC, Membrane attack complex; MCI, Mild cognitive impairment; MCP-1, Macrophage chemoattractant protein 1; M-CSF, Macrophage colony stimulating factor; MMP, Matrix metalloproteinase; MMSE, Mini Mental State Examination; MSC, Mesenchymal stem cell; MSC-CM, Mesenchymal stem cell conditioned medium; NE, Norepinephrine; NET, Neutrophil extracellular trap; NF, Nanofiber; NF-κB, Nuclear factor κB; NPY, Neuropeptide Y; OA, Osteoarthritis; OCN, Osteocalcin; OP, Osteoporosis; OPC, Oligodendrocyte precursor cell; OPG, Osteoprotegerin; OPN, Osteopontin; OS, Oyster shell; PAD4, Protein-arginine deiminase 4; PCL, Poly(ϵ-caprolactone); PDO, Polydioxanone; PLGA, Poly-lactic-co-glycolic acid; PLO, Poly-L-ornithine; PNS, Peripheral nervous system; RA, Rheumatoid arthritis; RAF, RAF proto-oncogene serine/threonine-protein kinase; RAGE, Receptor for advanced glycated end products; RANKL, Receptor activation of nuclear factor κB; REST, RE1-silencing transcription factor; ROS, Reactive oxygen species; SAN, Self-assembling nanofiber scaffold; SASP, Senescence-associated secretory phenotype; SERM, Selective estrogen response modulator; SF, Synovial fluid; SIRT1, Sirtuin 1; SP, Substance P; SYN, Alpha-synuclein; TCP, beta-tricalcium phosphate; TGF, Transforming growth factor; TH, Tyrosine hydroxylase; Th1, T helper subtype 1; TLR, Toll-like receptor; TNF, Tumor necrosis factor; Treg, Regulatory T-cell; TREM2, Triggering receptor on myeloid cells 2; VCAM, Vascular cell adhesion protein; VIP, Vasoactive intestinal peptide; YY1, Yin yang 1.
